# Effect of herbivore stress on transgene behaviour in maize crosses with different genetic backgrounds: *cry1Ab* transgene transcription, insecticidal protein expression and bioactivity against insect pests

**DOI:** 10.1186/s12302-023-00815-3

**Published:** 2023-11-28

**Authors:** André Felipe Lohn, Miluse Trtikova, Ignacio Chapela, Johnnie van den Berg, Hannalene du Plessis, Angelika Hilbeck

**Affiliations:** 1 Plant Ecological Genetics, Institute of Integrative Biology, Department of Environmental Systems Science, ETH Zürich, Zurich, Switzerland; 2 Department of Environmental Science, Policy and Management, University of California Berkeley, Berkeley, CA USA; 3 Unit for Environmental Sciences and Management, North-West University, Potchefstroom, South Africa

**Keywords:** Bt maize, Transgene flow, Herbivore feeding, *Helicoverpa armigera*, *Spodoptera littoralis*, Landrace, Open-pollinated varieties, Insect resistance management

## Abstract

**Background:**

Decades after their first commercial release, many theoretical assumptions are still taken for granted in the deployment of genetically modified (GM) crops. Theoretically, in the case of maize, active transcription of the *cry1Ab* transgene would result in dose-dependent production of the insecticidal Cry1Ab protein, which would in turn induce dose-dependent mortality on lepidopteran pests. We produced data to realistically approach this question by using a model that includes two genetic background contexts from two geographical provenances in Brazil and South Africa, and two lepidopteran pests (*Helicoverpa armigera* and *Spodoptera littoralis*). However, in this study, the effect of insect herbivory was superimposed to investigate possible stress-induced effects in transgene expression at three levels: mRNA, protein and bioactivity.

**Results:**

Overall, we found that herbivore damage by *H. armigera* was reflected only at the translational level, with a higher level of Cry1Ab protein measured in the Brazilian crosses under herbivore stress. On the other hand, compared to non-stress growing conditions, the herbivore damage by *S. littoralis* was not directly reflected in mRNA, protein or bioactivity in the South African crosses.

**Conclusions:**

The differences between South African and Brazilian genetic backgrounds, and between the stressor effect of the two herbivores used, highlight the complexity of transgene expression at the agroecological level.

**Supplementary Information:**

The online version contains supplementary material available at 10.1186/s12302-023-00815-3.

## Background

Genetically Modified (GM) crops, have been commercially grown for over a quarter-century on large acreages in a variety of geographical locations [1]. The assumption underlying both the development and cultivation of these crops has been that biotic and abiotic stresses have little influence on the performance of the desired traits introduced through transgenic manipulation, which would remain robust and efficacious under a variety of conditions. In other words, the introduction of a transgene into the genome of an unrelated recipient crop plant should theoretically result in, firstly, reliable and constant levels of its expression through transcription into mRNA, and subsequently a correlation between transgene transcription activity and the amount of the protein translated (here, Bt toxin). Finally, the amount of translated Bt toxin, and the expression of the bioactivity of that toxin (here, the killing of targeted pests) should also correlate with the respective mRNA production- a three-way relationship. Such three-way relationships were rarely subject of experimental investigations in the past, with research mostly reducing this complex relationship to mRNA serving as proxy for protein production, i.e. that transcript abundances are the main determinant of protein abundances, with implicit assumptions/expectations regarding the role of the produced proteins [2]. The hope was that if “*RNA levels could be used to predict protein levels, the value of these extensive expression resources would substantially increase, thereby allowing protein level prediction studies based on genome-wide transcriptomics data tremendously benefit systems biology efforts of human biology and disease*.” [3]. However, most studies had mixed outcomes and were mainly carried out in the field of human biology and often for specific medical conditions, for example related to cancer research [3, 4]. Despite the mixed outcomes, there remain the assumptions that neither genetic context (i.e. the rest of the genome beyond the transgenic DNA sequences) nor ecological conditions should, according to current theory, affect the performance of the GM crop and its expressed GM trait in a significant manner. While some of the evidence on the performance of GM crops, which is generally derived from highly controlled environments under irrigation, fertilization and intensive chemical inputs [5], appears to support these assumptions, little is known about these three-way interactions following gene flow into genetically heterogenous varieties grown under stressful conditions.

In the past, only two-way relationships were studied, at best, for one of the two main classes of GM crops dominating cultivation globally, namely insect-resistant GM crops expressing the transgene from *Bacillus thuringiensis* (Bt). Studies across different Bt crop plants have found that there are not only temporal and spatial variations in the concentration of the Bt insecticidal protein [6–10], but also that environmental stress factors such as temperature, salinity, waterlogging, drought and CO_2_, can induce Bt plants to produce lower Bt toxin concentrations [11–24]. Stress-dependent changes in Bt toxin levels have also been shown not to always correlate with changes in transgene expression [25], and the efficacy of Bt crops cannot always be explained by variations in Bt protein content [15, 23, 24, 26]. The introduction of an actively transcribing DNA sequence from *B. thuringiensis* into the plant genome and the resulting production of insecticidal Cry1Ab protein, has been useful in exploring transgene expression not only because this is the second most important GM trait, but also because it depends on the simple direct relationship outlined above: transgene-mRNA-protein-bioactivity. Maize (*Zea mays*) is one of the three main crops subject to the Bt-transgene intervention. Here, we present evidence of the complexities brought about by the genomic context of maize into which the Bt transgene is introduced, and which, in tandem with herbivore stressors, generate unexpected transgene-to-bioactivity relations. We focus on rarely studied materials, i.e. open-pollinated varieties, from relatively marginal agronomic conditions in South Africa and Brazil, which nonetheless represent a large proportion of those agroecological situations into which GM-crops are currently expanding.

Most studies in this field have focused on abiotic stressors and, to our knowledge, few studies have investigated the effects of biotic stress factors such as herbivory, on the efficacy of the Bt trait. In addition, few studies have addressed the way in which different environmental factors and genetic backgrounds may modulate the three-way interaction between (trans) gene transcription, protein levels and bioactivity. Most prior research focused only on two-way relationships, either transgene expression and protein concentration or protein concentration and bioactivity of the trait [6–11, 13, 15–20].

All in all, the theoretical assumptions necessary for a predictable performance of GM genetics in the field have long been overdue for empirical evaluation. In order to contribute to this necessary empirical evaluation, we have endeavoured to provide tests of theoretical assumptions by measuring transgene function at three levels: gene transcription rates (specific mRNA levels), protein expression (Cry1Ab concentration in leaf tissues), and bioactivity (insect mortality) in various crosses between GM and non-GM varieties under biotic stress conditions.

## Material and methods

We used a model that was previously described by Lohn et al. [27]*,* which includes two genetic background contexts from Brazil and South Africa, and two lepidopteran pests, *Helicoverpa armigera* (Lepidoptera: Noctuidae) and *Spodoptera littoralis* (Lepidoptera: Noctuidae). In this study, however, the effect of insect herbivory was superimposed to study possible stress-induced effects in transgene expression at three levels: mRNA, protein and bioactivity.

### Plant material and climate chamber conditions

The plant materials and setup of these experiments followed closely those of similar experiments carried out under non-stress growing conditions as described by Lohn et al. [27]. All materials and methods used in this paper match those in Lohn et al. [27], except that here the maize plants from different crosses were exposed to biotic stress (i.e. feeding damage) exerted by two different herbivorous pests of maize, *H. armigera* and *S. littoralis*. Maize plants were cultivated in a climate chamber at ETH Zürich (Kälte 3000 AG, Switzerland), under the same climate conditions and with the same numbers of seedlings per genetic background as described in Lohn et al. [27] (Additional file 2: Table S1).

**Fig. 1:**
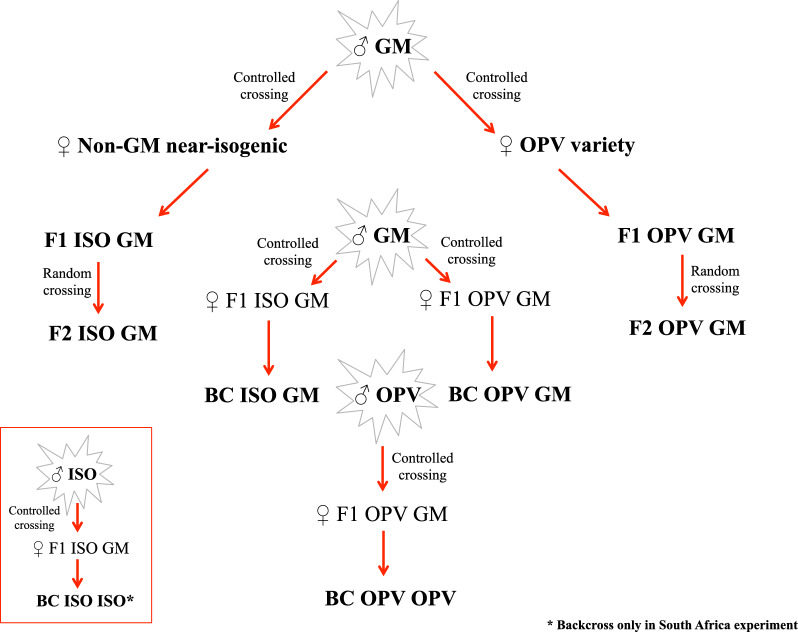
Diagram indicating how the crosses and backcrosses from Brazil and South Africa were obtained [27]

Crosses and successive backcrosses were performed under field conditions in Brazil and South Africa, as described in Lohn et al. [27] (Fig. 1). The Brazilian varieties used were Bt maize (MON810/AG5011YG, called GM here), non-Bt near-isogenic maize (AG5011, called ISO here) and the open-pollinated variety Pixurum 5 (called OPV here). The plant material from South Africa consisted of seeds from Bt maize (MON810/PAN 6Q 308B, called GM here), non-Bt near-isogenic maize (PAN 6P-110, called ISO here) and an OPV named Kalahari. The F1 crosses of OPV x Bt maize and the ISO x Bt maize were generated using the hybrid Bt maize as male plant and either the OPV or the non-Bt maize as female. The segregating F2 populations (called F2 OPV Bt and F2 ISO Bt) were formed from the random crossing of the above-described F1 plants. Two backcrosses (named BC) were produced by crossing the above-described F1 plants again with the male maize plants of their Bt parents (BC OPV Bt and BC ISO Bt). The BC ISO ISO was produced by backcrossing F1 ISO Bt plants with the male ISO parent maize again. Finally, the BC OPV OPV was produced by backcrossing F1 OPV Bt plants with the male OPV parent maize again. We tried to also obtain seeds from BC OPV OPV crosses, but this genetic background produced an insufficient amount of seeds for the experiments. Therefore, we chose to exclude this genetic background. In addition, limitations in plot space in the field in Brazil, coupled with time constraints to complete the work in Zurich made it impossible to include BC ISO ISO from Brazil.

### Insects

*Helicoverpa armigera* eggs were acquired from ENTOMOS AG (Switzerland) and maintained in growth chambers (Sanyo MLR 350, Japan). Some eggs were incubated for five days, at 26 °C, a photoperiod of 16:8 h (L:D) and light intensity of 20% (fluorescent lamp FL40SS W/37). Larvae were reared on a wheat-germ-based artificial diet [28] until they reached the fourth instar, after which they were transferred to feed on the experimental maize plants grown in pots. The other insect eggs were incubated for eight days, under the same conditions, but kept on artificial diet only until the second instar, after which they were used in mortality bioassays. *Spodoptera littoralis* larvae of the fourth and second instar were provided by Syngenta Crop Protection (Stein, Switzerland). Fourth-instars were placed onto potted maize plants to feed and induce herbivore damage while second-stars were used in the mortality bioassays.

According to the relevant literature, *H. armigera* and *S. littoralis* are considered non-target insect pests of Bt maize, although they are sensitive to the Cry1Ab protein [29–31]. Indeed, first and second-instar larvae of *S. littoralis* have been reported to be highly susceptible to Bt toxins [32], with a gradual reduction in susceptibility in later instars [33].

In Brazil, the emergence of the *H. armigera* larvae as a pest was confirmed in 2013 [34], and already in the first cropping season thereafter, losses of approximately USD 0.8 billion were ascribed to this pest [35]. *Spodoptera littoralis* occurs throughout Africa and, while it attacks several crop species, it is most important on cotton [36–38].

### Stress experiments with insect herbivores

Herbivore damage on the Brazilian crosses was caused by placing five *H. armigera* larvae (mostly fourth-instar) on each maize plant. For the South African crosses, the herbivore damage was caused by placing ten *S. littoralis* larvae on each maize plant (mostly fourth instar). All maize plants were between the V5 and V7 growth stages during inoculation with larvae. Since the first instars of these species are highly susceptible to the Cry1Ab protein, fourth-instars were used to inflict leaf feeding damage to plants, and, in doing so, induce stress to these plants. To prevent escape of the larvae into neighbouring plants, each maize plant was covered with a Plexiglas® cylinder (80 cm high/18 cm diameter) and the gap between saucer and pot was sealed with tape (Additional file 1: Fig. S1). After three days of feeding by the *H. armigera* larvae and seven days by the *S. littoralis* larvae, leaf samples were collected to determine transgene transcription (mRNA) using qRT-PCR, while Cry1Ab protein concentration was determined using ELISA (below). Larval mortality rates were also recorded.

### Molecular and bioactivity analyses

The *cry1Ab* transgene expression, Cry1Ab concentration and bioactivity levels were analyzed using methods previously described by Lohn et al. [27]. These analyses are briefly described below.

Relative abundance of mRNA transcripts corresponding to the *cry1Ab* transgene was established through reverse-transcriptase, real-time polymerase chain reaction assays (qRT-PCR). mRNA in leaf samples was extracted with a standard kit (NucleoSpin®, Macherey–Nagel, Germany), and after quantitative standardization and purity control, utilized in triplicate for cDNA synthesis (QuantiTect® Reverse Transcription Kit; Qiagen®, Germany). cDNA was used for PCR amplification of *cry1Ab* transgene sequences, as well as three reference gene sequences (*mep*, *ubcp*, *lug*; Manoli et al. [39]) using primers and conditions described in Lohn et al. [27]. The values reported are in relative units, comparing PCR amplification efficiency (Ct values) of the normalized control reference genes to that of the *cry1Ab* transgene sequences, as detailed by Lohn et al. [27] and Bookout and Mangelsdorf [40].

For evaluation of translational activity, the concentration of Cry1Ab protein was estimated for the same leaf samples used for mRNA evaluation, using an Enzyme-Linked Immunosorbent Assay (ELISA). Quantitative extraction of protein (FastPrep-24; MP Biomedicals, Germany) was followed by specific quantification of the Cry1Ab protein using a Double Antibody Sandwich ELISA kit (Agdia®, USA), with the aid of an external calibration curve based on standards of purified Cry1Ab.

Bioactivity of the *cry1Ab* transgene products was evaluated by determining the larval mortality/survival rates after feeding on test plants. These insect species have medium-level susceptibility to the Cry1Ab protein (see below), allowing for quantitative results to be obtained from rates of survival after exposure to plant materials containing the toxin. Insects were reared on artificial diet until second-instar, as detailed by Lohn et al. [27]. Larvae were fed ad libitum on leaf material from 6 to 8 plants of the appropriate cross/treatment, and the number of surviving larvae determined after four days. Materials and conditions for these bioassays are described in detail by Lohn et al. [27].

The experimental setup was carried out in four replicates with 1–2 plants from each genetic background in each replicate. In total for all four replicates, 6–8 plants from each genetic background were used for the different analyses (Additional file 2: Table S1). The maize plants were in the V5 and V7 stage and, for each plant, the fifth fully developed leaf was sampled. Six circular leaf samples (∅ 1.5 cm) were cut out with a cork-cutter, three on each side of the central vein of the leaf but avoiding the central leaf vein. Four leaf disks were designated for transgene expression analysis and two leaf disks for quantification of the Cry1Ab concentration. More details were described by Lohn et al. [27]

### Statistics and computational analyses

The same statistical analyses of qRT-PCR, ELISA, bioassays and correlations were performed as described and Lohn et al. [27]. Analyses were conducted in R [41] and the figures were produced using the package ggplot2 [42]. For multiple comparison analyses, we used the packages lsmeans and multcomp [43, 44]. In summary, when in an isolated factor (different genetic backgrounds or different levels of herbivore damage) were found significant differences in the respective analyses (*P* < 0.05) the Duncan test was applied. However, when the interaction effects between the two factors were found to be significant (*P* < 0.05), Least Square Means analyses were applied.

## Results and discussion

### Transgene transcription levels

**Fig. 2:**
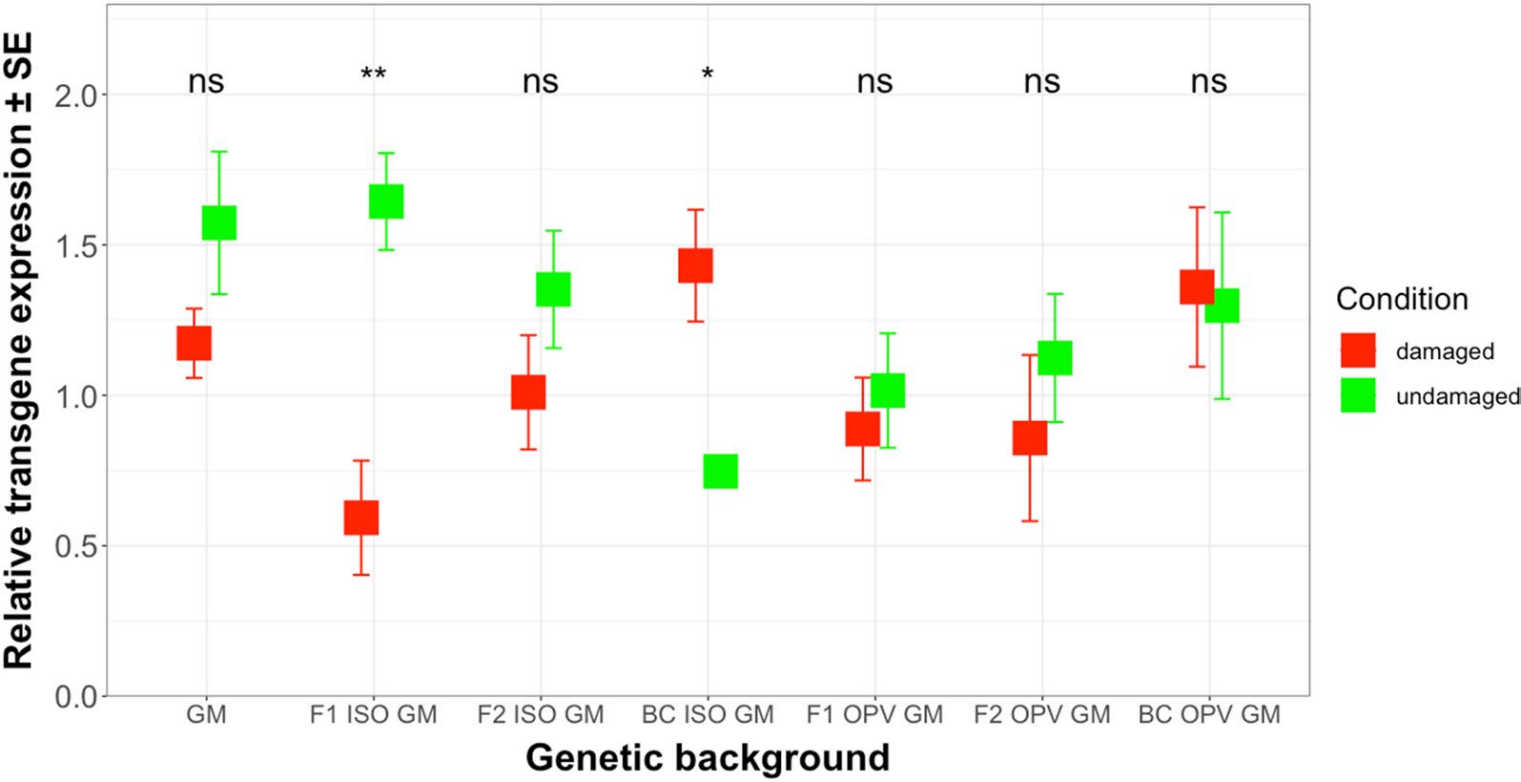
Mean relative transgene expression in damaged and undamaged plants of different genetic backgrounds from Brazil. The values reported are in relative units. The feeding damage was induced by *H. armigera* larvae. Vertical bars show mean values, and the standard errors (± SE) are indicated as lines. The transgene transcription level was compared in each different genetic background between damaged and undamaged conditions. *P < 0.05, **P < 0.01, ns: not significant

The mean transgene transcription level (relative mRNA level) in leaves of plants subjected to damage by *H. armigera* ranged from 0.59 (relative units) in F1 ISO GM to 1.43 in BC ISO GM for the different genetic backgrounds from Brazil (Fig. 2) (Additional file 3: Table S2). No significant difference in transgene transcription levels could be observed between the different genetic backgrounds (*F* = 1.204; df = 6; *P* = 0.312) or between different levels of herbivore damage (*F* = 2.807; df  = 1; *P* = 0.100). However, there was a significant interaction effect between the different genetic backgrounds and the different levels of herbivore damage on mRNA level (*F* = 2.833; df = 6; *P* < 0.05). In other words, when factors were analyzed separately (different genetic backgrounds and levels of herbivore damage), there was no significant difference in the transgene transcription levels, but we found a significant difference at the mRNA level when the interaction effect between the respective factors was analyzed. Further, Least Square Means analyses for multiple comparisons revealed that the transgene transcription level differed significantly between damaged and undamaged plants of the F1 ISO GM cross (*t* ratio = − 3.123; df = 85; *P* < 0.01) and in the BC ISO GM backcross (*t* ratio = 2.314; df = 85; *P* < 0.05). However, the results did not follow a consistent pattern, since the damage inflicted by *H. armigera* caused a significant decrease (more than 2.7-fold) in the transgene transcription level in F1 ISO GM, while it caused a significant increase (more than 1.9-fold) in the BC ISO GM, which also exhibited the two most distant mean expression values (Fig. 2). In addition, *H. armigera* damage did not affect transgene transcription levels in any other genetic background compared to those in maize plants under undamaged conditions, including the GM parental maize (Fig. 2).

**Fig. 3:**
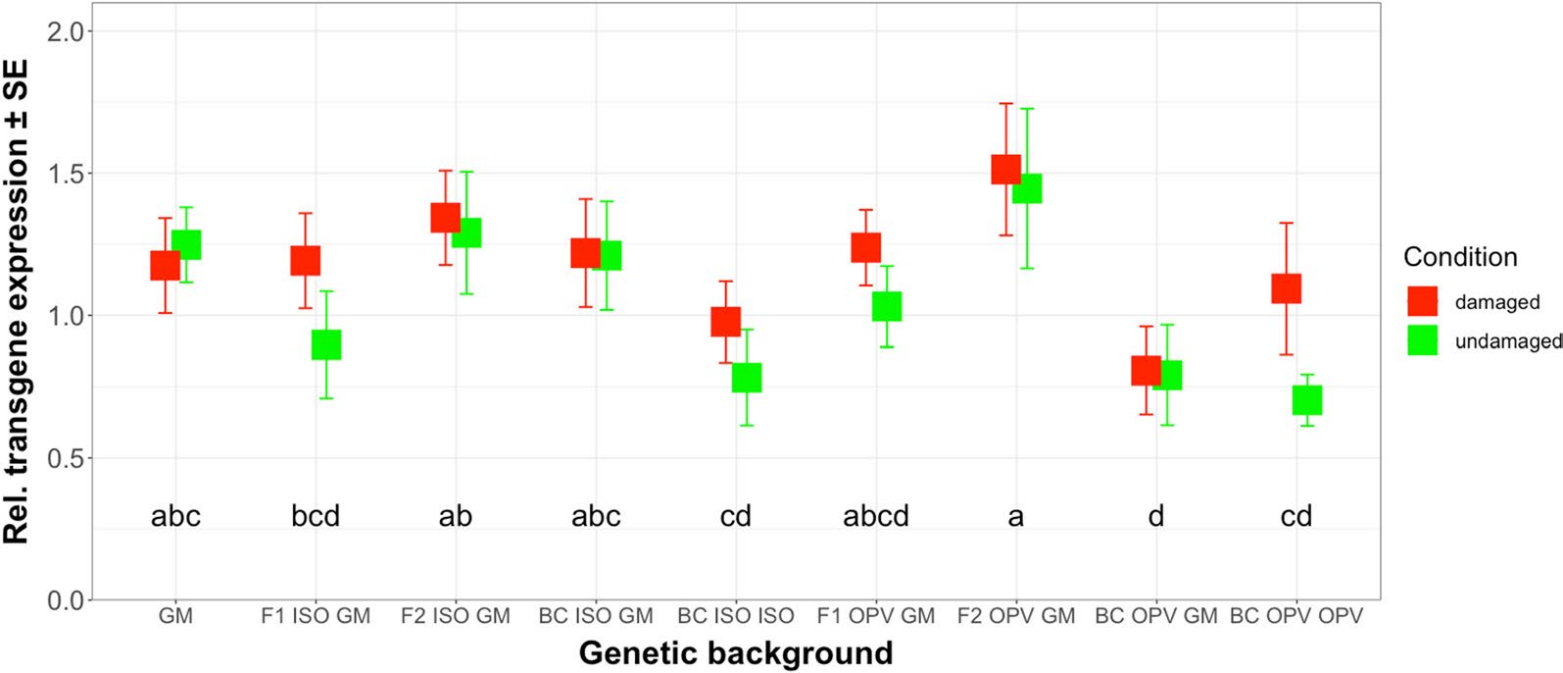
Mean relative transgene expression in damaged and undamaged plants of different genetic backgrounds from South Africa. The values reported are in relative units. The feeding damage was induced by *S. littoralis* larvae. Vertical bars show mean values, and the standard errors (± SE) are indicated as lines. Letters above the bars represent results from the means separation Duncan’s test

Transgene transcription levels under herbivore damage by *S. littoralis* in different genetic backgrounds from South Africa ranged from 0.80 in BC OPV GM to 1.51 in F2 OPV GM (Fig. 3) and (Additional file 3: Table S2). In the respective crosses, we did not find significant differences in transgene transcription level between damaged and undamaged plants (*F* = 2.844; df = 1; *P* = 0.09) nor in the interaction between different genetic backgrounds and herbivore damage (*F* = 0.326; df = 8; *P* = 0.954). However, there was a significant difference in transgene transcription level between different genetic backgrounds (*F* = 2.840; df = 8; *P* < 0.01). A Duncan multiple range test showed that transcription levels were significantly higher in the F2 OPV GM cross than in the F1 ISO GM, BC ISO ISO, BC OPV GM and BC OPV OPV regardless of herbivore damage. Additionally, the transgene transcription level was significantly higher in the F2 ISO GM cross than in the BC ISO ISO, BC OPV GM and BC OPV OPV. When compared to the GM parental maize, only the BC OPV GM backcross exhibited significantly lower transcription levels. Overall, the F2 plants tended to show higher transgene expression (transcription activity) than other genetic backgrounds, possibly owing to these maize plants being homozygous for the transgene in these crosses. The formation of homozygous plants was also possible in the BC OPV GM crosses but this genetic background showed significantly lower transgene transcription levels compared to other genetic backgrounds that could have been expressing two copies of the transgene. However, the latter was not established since (trans) gene load was not determined in this study. All Duncan multiple range test results are shown in Fig. 3.

For further analysis, the different genetic backgrounds of Brazilian and South African crosses were pooled and grouped according to ISO or OPV parental lines, without differentiating between F1’s, F2’s and BC’s. These pooled data were renamed ‘ISO crosses’ and ‘OPV crosses’ and the results are described in Additional file 4: Table S3. The mean transgene transcription level in the leaves of the different Brazilian crosses subjected to herbivore feeding ranged between 1.00 in OPV crosses and 1.17 in GM parental maize (Additional file 4: Table S3). There was no significant difference in transcription level between the different groups (*F* = 1.585; df = 2; *P* = 0.210), conditions (*F* = 2.541; df = 1; *P* = 0.114) nor in the interaction between the different groups and conditions (*F* = 0.315; df = 2; *P* = 0.731). In the South African crosses, transgene transcription level under herbivore damage conditions ranged between 1.17 in GM parental maize and ISO crosses and 1.19 in OPV crosses (Additional file 4: Table S3). Again, there was no significant difference in transgene transcription level between the different groups (*F* = 0.421; df = 2; *P* = 0.657), conditions (*F* = 3.117; df = 1; *P* = 0.080) or interaction between the different groups and conditions (*F* = 0.514; df = 2; *P* = 0.600).

Analysis of mRNA measurements, which reflect transgene transcription levels, showed that *H. armigera* damage caused important but unpredictable effects in the Brazilian crosses. While herbivory by *H. armigera* larvae did not alter transcription level in most genetic backgrounds (in GM parental maize, F2 ISO GM, F1 OPV GM, F2 OPV GM and BC OPV GM), results showed decrease or increase in transcription level in F1 ISO GM and in BC ISO GM, respectively. In the South African crosses, damage by *S. littoralis* did not significantly alter transgene transcription levels. Additionally, when different genetic backgrounds were pooled according to ISO and OPV parental lines, the results did not show any effect of herbivore stress in either the Brazilian or the South African crosses.

Overall, the statistical analyses could not detect consistent changes in transgene transcription levels due to herbivory stress caused by *H. armigera* or *S. littoralis*, regardless of genetic background of the plants involved. This result is consistent with previous studies by Olsen et al. [26] who reported no significant change in *cry1Ac* transgene expression in response to *H. armigera* feeding damage to GM cotton leaves.

Little is known about the impact of herbivory on transgene expression (transcription activity) in GM plants, with only one main study dealing with GM cotton (Olsen et al. [26]). Other studies with non-engineered plants relate herbivore damage effects to alterations in defence-related genes, phytohormones, volatile organic compounds and secondary metabolism [45–55].

Abiotic stress factors can influence transgene transcription level of Bt maize plants. Trtikova et al. [25] reported that the *cry1Ab* transgene expression in Bt maize plants under cold/wet stress was similar to the expression under optimal conditions, but that it was significantly reduced under hot/dry stress. However, in another Bt maize variety, the *cry1Ab* transgene transcription levels measured as mRNA in leaves under cold/wet and hot/dry stresses were similar to levels in the control treatments. In GM petunia plants, transgene expression was reduced after exposure to high temperatures, possibly related to hypermethylation of the CaMV 35S promoter [56]. Li et al. [57] reported that NaCl stress significantly increased *cry1Ac* mRNA transcript levels in two GM cotton cultivars. In summary, results from our study concur with prior reports showing that different types of biotic and abiotic stress factors may influence transgene transcription levels, but that the direction of this response is currently unpredictable.

### Cry1Ab concentration

**Fig. 4:**
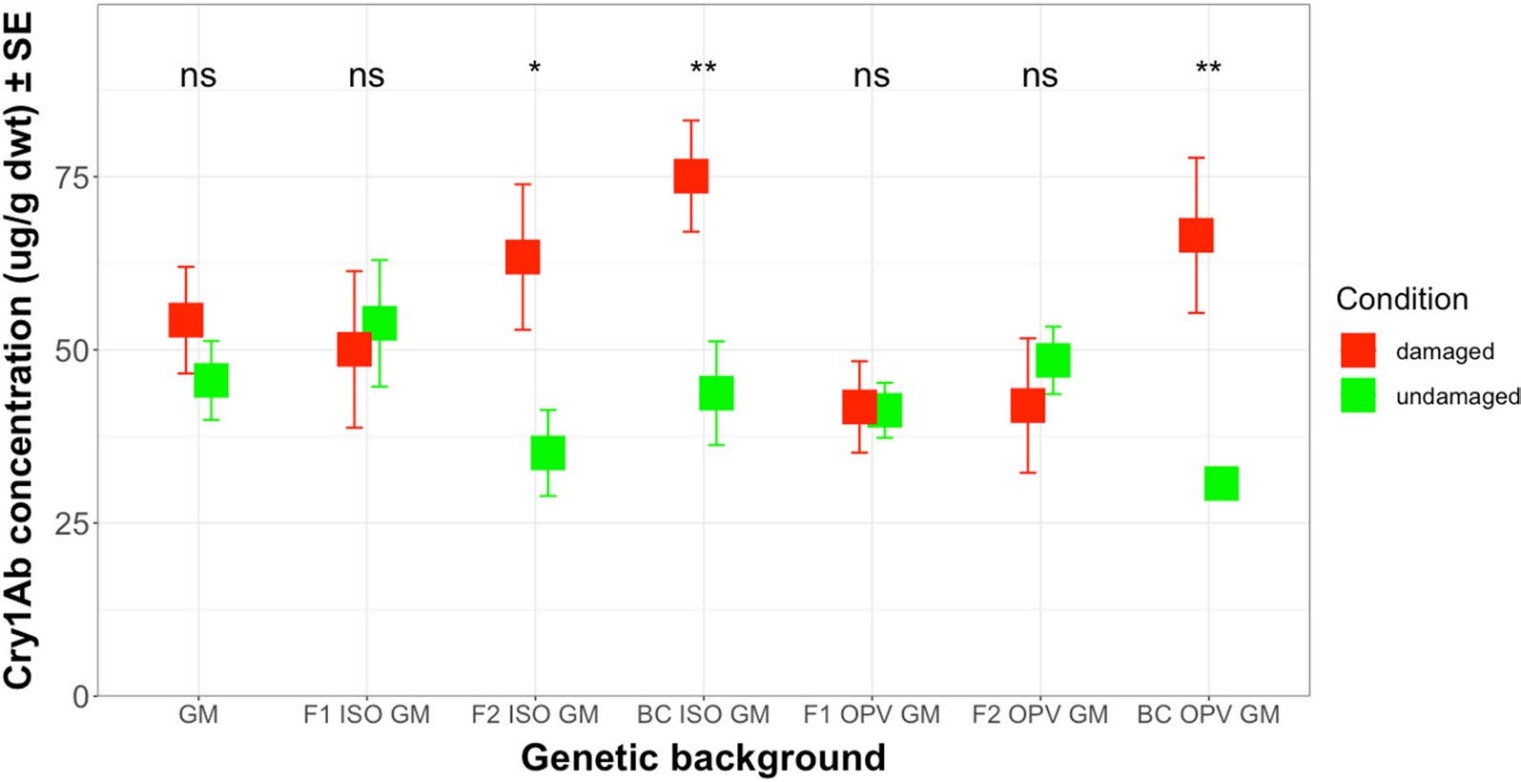
Mean Cry1Ab concentration (µg/g dwt) in damaged and undamaged plants of different genetic backgrounds from Brazil. The feeding damage was induced by *H. armigera* larvae. Vertical bars show mean values, and the standard errors (± SE) are indicated as lines. The Cry1Ab concentration was compared in each different genetic background between damaged and undamaged conditions. *P < 0.05, **P < 0.01, ns: not significant

The mean concentration of the insecticidal protein Cry1Ab in maize leaves of different genetic backgrounds from Brazil, damaged by *H. armigera,* ranged from 41.76 µg/g dwt (dry weight tissue) in F1 OPV GM to 75.08 µg/g dwt in BC ISO GM (Fig. 4) and (Additional file 5: Table S4). Mean concentration of Cry1Ab toxin in undamaged plants only ranged from 35.12 (µg/g dwt) in F2 ISO GM to 48.48 µg/g dwt in F2 OPV GM (Additional file 5: Table S4). Similarly, results for the South African maize genotypes exposed to herbivory by *S. littoralis* showed a mean Cry1Ab concentration ranging from 23.29 µg/g dwt in F2 OPV GM to 38.25 µg/g dwt in BC OPV GM, while the corresponding mean concentration of Cry1Ab toxin in undamaged plants ranged from 20.40 µg/g dwt in F1 OPV GM to 40.85 µg/g dwt in GM parental maize (Additional file 5: Table S4).

In the Brazilian crosses, plants from different genetic backgrounds subjected to herbivore-induced damage produced significantly higher concentrations of Cry1Ab toxin than undamaged plants, regardless of the genetic background (*F* = 11.901; df = 1; *P* < 0.01). However, a significant interaction effect between genetic backgrounds and the presence or absence of herbivory was also observed (*F* = 3.191; df = 6; *P* < 0.01). Least Square Means for multiple comparisons revealed that the Cry1Ab concentration differed significantly between stressed and undamaged plants in F2 ISO GM (*t* ratio = 2.557; df = 78; *P* < 0.05), as well as the backcrosses BC ISO GM backcross (*t* ratio = 3.603; df = 78; *P* < 0.01) and BC OPV GM (*t* ratio = 3.058; df = 78; *P* < 0.01). Damage caused by *H. armigera* larvae resulted in a significant increase (ca. 1.6–1.8-fold) in Cry1Ab concentrations in these crosses. However, the damage caused by *H. armigera* feeding did not significantly affect Cry1Ab concentrations in other genetic backgrounds, although it tended to be somewhat higher, except for the F2 OPV GM cross (Fig. 4).

**Fig. 5:**
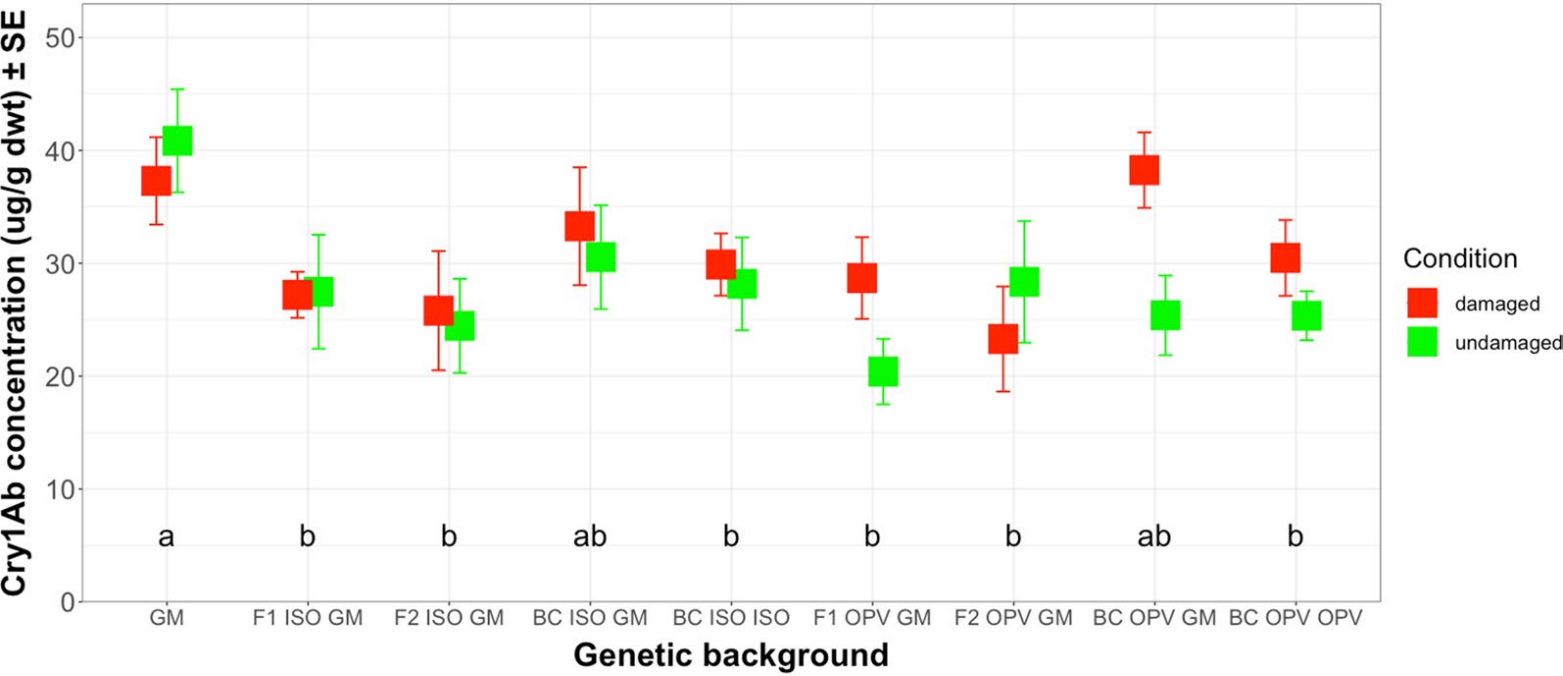
Mean Cry1Ab concentration (µg/g dwt) in damaged and undamaged plants of different genetic backgrounds from South Africa. The feeding damage was induced by *S. littoralis* larvae. Vertical bars show mean values, and the standard errors (± SE) are indicated as lines. Letters above the bars represent results from the means separation Duncan’s test

For South African maize materials under herbivore stress, mean Cry1Ab concentration in plants damaged by *S. littoralis* ranged from 23.29 µg/g dwt in F2 OPV GM to 38.25 µg/g dwt in BC OPV GM, while the corresponding values for undamaged plants ranged from 20.40 µg/g dwt in F1 OPV GM to 40.85 µg/g dwt in GM parental maize (Fig. 5) and (Additional file 5: Table S4). Here, ANOVA tests showed differences compared with Brazilian crosses, since significant differences in Cry1Ab concentrations for South African materials appeared only between different genetic backgrounds (*F* = 2.082; df = 8; *P* < 0.05). The test did not show significant differences in Cry1Ab concentrations between damaged and undamaged plants (*F* = 2.240; df = 1; *P* = 0.137), nor did it support an interaction between genetic backgrounds and the different growing conditions such as those observed in Brazilian backgrounds (*F* = 0.974; df = 8; *P* = 0.460). Interestingly, Cry1Ab concentrations for the South African materials were significantly higher in the GM parental maize plants than in many of the different genetic backgrounds. Such is the case in F1 ISO GM, F2 ISO GM. BC ISO ISO, F1 OPV GM, F2 OPV GM and BC OPV OPV. Statistically, only the plants in BC ISO GM and BC OPV GM exhibited similar Cry1Ab concentrations as the GM parental maize plants (Fig. 5).

Overall, across the different South African crosses, results did not show significant differences in mean Cry1Ab concentrations between plants that suffered herbivory and those left undamaged. Maize plants produced 30.43 µg/g dwt when damaged and 27.69 µg/g dwt (Additional file 6: Table S5) when left undamaged.

For further analysis, different crosses from Brazil and South Africa were pooled and grouped into ISO or OPV parental lines, without distinction between F1’s, F2’s and BC’s. These groups of pooled data were renamed ‘ISO crosses’ and ‘OPV crosses’ and the results are described in Additional file 7: Table S6.

**Fig. 6:**
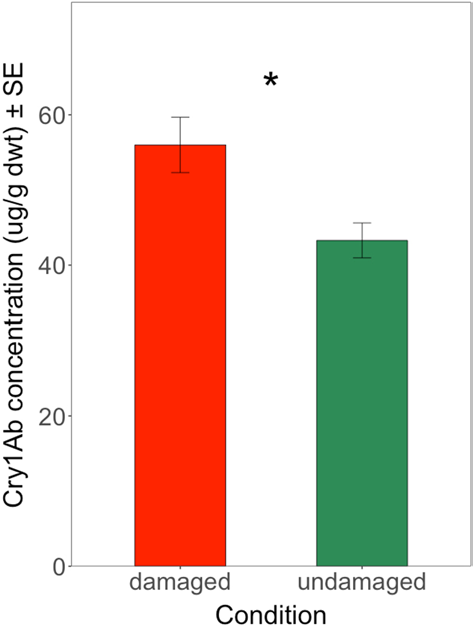
Mean Cry1Ab concentration (µg/g dwt) in undamaged and damaged across all different genetic backgrounds from Brazil. The feeding damage was induced by *H. armigera* larvae. Vertical bars show mean values, and the standard errors (± SE) are indicated as lines. *P < 0.05 (ANOVA test)

Mean Cry1Ab concentrations in leaves of the different crosses from Brazil ranged from 48.59 µg/g dwt to 64.00 µg/g dwt in the herbivore damaged OPV and herbivore damaged ISO crosses, respectively. Mean Cry1Ab concentrations in herbivore damaged GM parent plants were between those of damaged ISO and OPV crosses (54.29 µg/g dwt). In the undamaged control plants, the mean Cry1Ab concentrations of these different crosses from Brazil were always lower than < 44 µg/g dwt (Additional file 7: Table S6). The ANOVA test revealed a significant difference in Cry1Ab concentrations between the different growing conditions (*F* = 4.735; df = 1; *P* < 0.05) but not between the different GM plant groups (*F* = 1.125; df = 2; *P* = 0.329) or the interaction between the different GM plant groups and the different growing conditions (*F* = 0.513; df = 2; *P* = 0.600). Overall, *H. armigera*-induced damage in the maize crosses from Brazil led consistently to higher Cry1Ab protein concentrations (55.99 µg/g dwt) than in undamaged maize plants (43.29 µg/g dwt) (Fig. 6). This difference corresponded to a > 1.2-fold increase in production of Cry1Ab protein concentration.

**Fig. 7:**
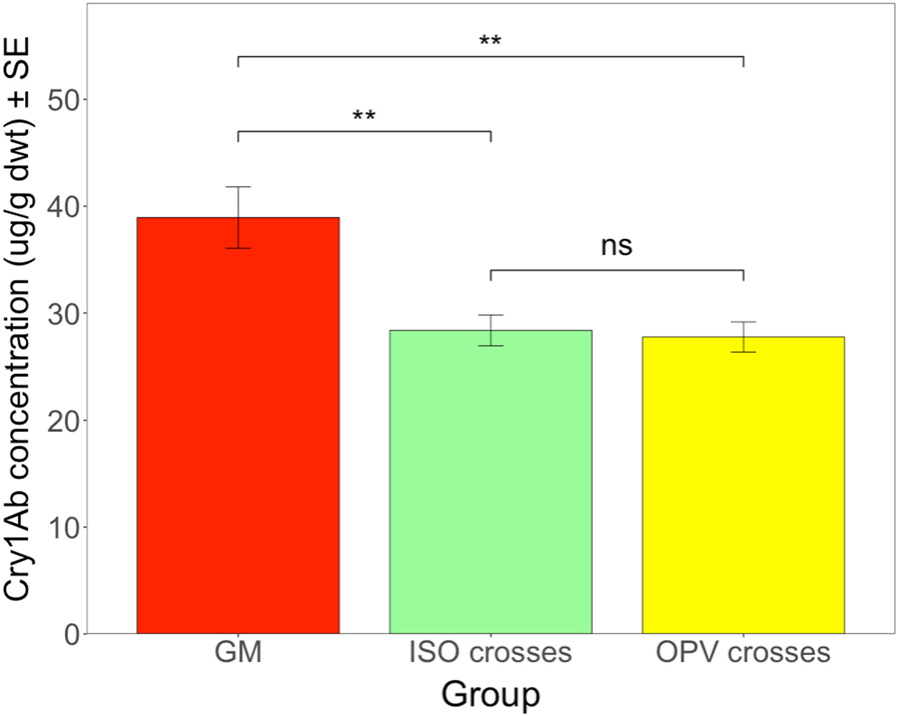
Mean Cry1Ab concentration (µg/g dwt) in different groups from South Africa. The feeding damage was induced by S*. littoralis* larvae. Vertical bars show mean values, and the standard errors (± SE) are indicated as lines. **P < 0.01, ns = not significant (Pairwise comparisons)

For different crosses from South Africa subjected to herbivory by *S. littoralis* larvae, mean Cry1Ab concentration ranged between 29.14 µg/g dwt in ISO crosses and 37.30 µg/g dwt in GM parental maize plants (Additional file 7: Table S6). The mean Cry1Ab concentration in herbivore-damaged OPV crosses was 30.18 µg/g dwt, compared to 25.17 µg/g dwt in undamaged OPV crosses and 40.85 µg/g dwt in undamaged GM parental plants (Additional file 7: Table S6). The ANOVA test revealed a significant difference in Cry1Ab concentrations between different GM plant groups (*F* = 5.045; df = 2; *P* < 0.01) but not between different growing conditions (*F* = 2.028; df = 1; *P* = 0.157) or the interaction between different GM plant groups and different growing conditions (*F* = 0.783; df = 2; *P* = 0.459). Pairwise comparisons between different groups from South Africa revealed a significant difference in Cry1Ab concentration between the GM parental maize and ISO crosses (*t* ratio = 2.982; df = 126; *P* < 0.01) and between the GM parental maize and OPV crosses (*t* ratio = 3.184; df = 126; *P* < 0.01) (Fig. 7). GM parental maize plants produced significantly higher Cry1Ab concentrations than the ISO and OPV crosses, regardless of herbivore damage. The analysis did not show a significant difference between the ISO crosses and OPV crosses (*t* ratio = 0.328; df = 126; *P* = 0.743) (Fig. 7). Taken together, these results revealed that the feeding damage inflicted by *S. littoralis* did not lead to higher Cry1Ab protein concentrations in maize crosses from South Africa stressed by herbivory, as was the case for the maize crosses from Brazil with herbivore damage by *H. armigera* (Fig. 6).

A Fligner-Killeen test applied to crosses from Brazil revealed a significant difference between the variance of Cry1Ab concentrations in damaged and undamaged GM plants (*P* < 0.01). In *H. armigera*-damaged maize plants, Cry1Ab concentration varied tenfold (between 11.8 µg/g dwt and 120.7 µg/g dwt), while in undamaged maize plants the Cry1Ab concentration varied only 4.4-fold, between 17.9 µg/g dwt and 78.6 µg/g dwt. Herbivore-induced damage did not only increase the Cry1Ab protein concentration in damaged maize plants but also led to much more variability in the measured concentrations than in undamaged maize plants.

In contrast, when applied to GM crosses from South Africa, the Fligner-Killeen test showed a non-significant difference in the variability in Cry1Ab protein concentration between damaged and undamaged plants (*P* = 0.690). In *S. littoralis*-damaged maize plants, the Cry1Ab concentration varied eightfold (between 7.5 µg/g dwt and 62.3 µg/g dwt) and tenfold in undamaged plants (between 5.1 µg/g dwt and 54.8 µg/g dwt).

Some researchers have speculated that the ability of Bt plants to produce Cry1Ab protein may be correlated with their efficiency in use of the amino acid precursors produced via photosynthesis and the nitrogen concentration available within the plants [58, 59]. In the latter case, herbivore damage would decrease the production of Cry1Ab protein by allocating the precursors and/or nitrogen for the production of defensive compounds in maize plants. However, the results of this study do not support this observation. Other studies have also reported varied effects of biotic stress resulting from herbivore damage on Bt toxin concentrations in GM plants. Olsen et al. [26], using GM Bt cotton plants, reported unchanged Cry1Ac toxin concentrations in undamaged and damaged plants fed upon by *H. armigera* larvae, while Prager et al. [60] reported that Bt maize plants infested with herbivorous spider mites exhibited lower Cry1Ab and Cry3Bb1 toxin concentrations than non-infested plants.

Abiotic stress, such as those that could result from extreme temperatures, water deficit, salinity, high irradiance, elevated CO_2_ and nutrient deficiency, can also influence Cry toxin concentrations in Bt plants. Chen et al. [11] found that the Cry1A concentrations decreased when Bt cotton plants were exposed to high temperature. Chen et al. [61] showed that the interaction between high temperature and extreme relative humidity caused a decrease of Cry1Ac concentrations. Parimala and Muthuchelian [16] also reported a reduction of Cry1Ac and Cry2Ab toxins in Bt cotton under high irradiance stress, while elevated CO_2_ concentration decreased the N allocation to Cry1Ac toxin production [58]. By contrast, in other studies, the increase of nitrogen fertilization resulted in an increase of the Bt protein production in GM cotton [62], in GM maize [63] and in GM rice plants [64]. Furthermore, other studies reported that NaCl stress led to a reduction of the Bt toxin concentration in GM cotton plants [13, 22, 23, 65]. Luo et al. [24] found that waterlogging and the combination of waterlogging and salinity reduced of the Cry1Ac toxin concentration in GM cotton plants. In a study with maize plants, Trtikova et al. [25] showed that Cry1Ab protein concentrations were similar in plants grown under optimal and hot/dry growing conditions, but that Bt maize plants exposed to cold/wet stress conditions had significantly higher Cry1Ab toxin concentrations than plants grown under optimal conditions. Based on such prior reports and our own results, we conclude that biotic and abiotic stresses can affect Cry1Ab protein production but not always, and if it does, the effect is unpredictable, and dependent of many factors, such as crop species and the type of stress factors.

### Correlation between transgene transcription and Cry1Ab concentration

**Fig. 8:**
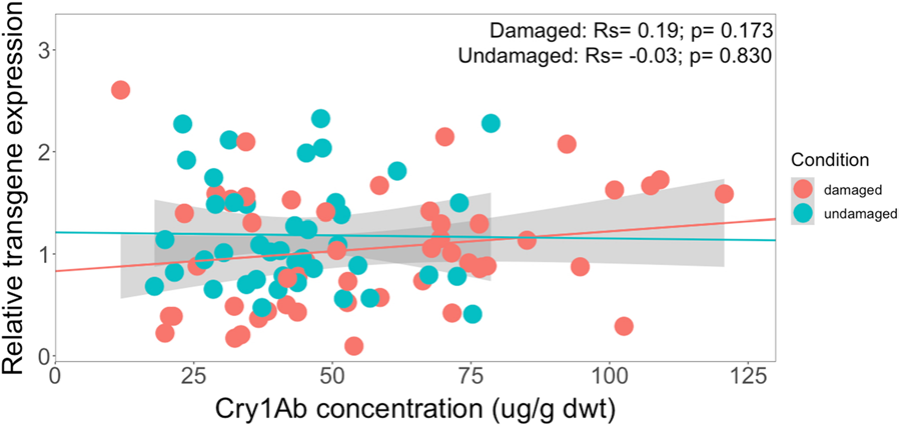
Spearman’s rank correlation coefficient (*Rs*) between transgene transcription activity (relative units) and Cry1Ab concentration (µg/g dwt) in damaged and undamaged plants across different genetic backgrounds from Brazil. The feeding damage was induced by *H. armigera* larvae. Damaged maize plants (*Rs* = 0.19, *P* = 0.173); undamaged maize plants (*Rs* = − 0.03, *P* = 0.830)

No significant correlation was observed between the measured Cry1Ab concentration and transgene transcription level across different Brazilian GM crosses subjected to herbivore feeding damage (*Rs* = 0.19, *P* = 0.173) (Fig. 8). These results were similar to those with undamaged GM maize plants. Although there was no significant correlation, Fig. 8 does show a larger variability of measured Cry1Ab concentrations with the highest Cry1Ab protein concentrations found in maize plants damaged by *H. armigera* larvae.

**Fig. 9:**
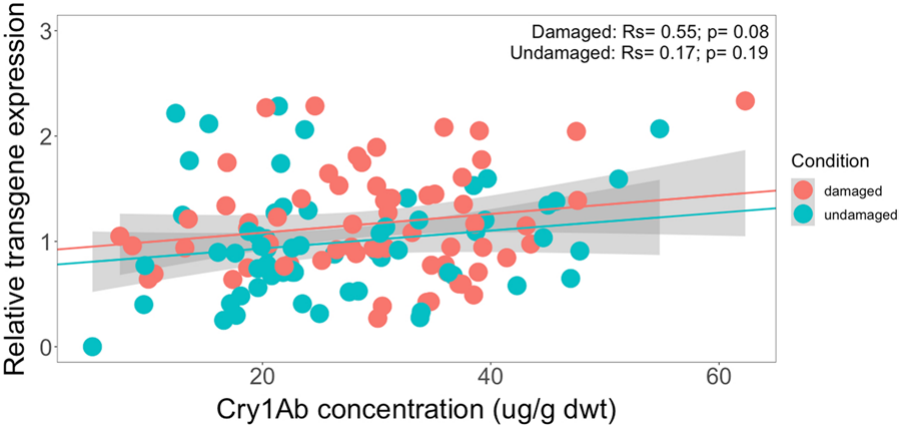
Spearman’s rank correlation coefficient (*Rs*) between transgene transcription activity (relative units) and Cry1Ab concentration (µg/g dwt) in damaged and undamaged plants across different genetic backgrounds from South Africa. The feeding damage was induced by *S. littoralis* larvae. Damaged maize plants (*Rs* = 0.55, *P* = 0.08); undamaged maize plants (*Rs* = 0.17, *P* = 0.190)

For the different crosses from South Africa, there was also no statistically significant correlation between Cry1Ab concentration and transgene transcription level, neither under herbivore damage (*Rs* = 0.55, *P* = 0.08), nor for undamaged GM maize plants (Fig. 9). However, Fig. 9 does illustrate that measured Cry1Ab concentration values were much more homogenously spread across both groups than it was for the Brazilian crosses.

When separating the different genetic backgrounds from Brazil and South Africa, results showed no correlation between Cry1Ab concentration and transgene transcription level under herbivore-induced conditions (Additional file 8: Table S7). When analysing the data grouped according to ISO or OPV genetic backgrounds, regardless whether from Brazil or South Africa, there was also no correlation between Cry1Ab concentration and transgene transcription level in any group (Additional file 9: Table S8).

These findings are in agreement with those of Trtikova et al. [25] who also reported a lack of correlation between the values for *cry1Ab* transgene expression and Cry1Ab protein concentrations in one Bt maize variety exposed to abiotic cold/wet or hot/dry stress. However, in the same study, another Bt maize variety under optimal growing conditions revealed a correlation between *cry1Ab* transgene expression and Cry1Ab protein concentration. But there was no correlation when the plants were exposed to cold/wet or hot/dry stress conditions, which the researchers attributed to Cry1Ab protein content being affected by the plant’s own regulatory system together with external environmental conditions. In a study with GM cotton plants, Cry1Ac mRNA transcript levels were increased in plants exposed to NaCl stress while Cry1Ac protein concentrations remained unaffected [57].

While our results revealed no differences in transgene transcription levels under herbivore damage by *H. armigera*, compared to undamaged conditions, the Cry1Ab protein concentration increased 1.2-fold compared to undamaged conditions nevertheless. A possible explanation could be that herbivore damage by *H. armigera* larvae acts at the translational regulatory system. While *H. armigera* feeding damage led to an increase in Cry1Ab concentrations in almost all GM crosses, this increase was not clearly correlated with the activity of the transgene, as measured by transcription levels of the corresponding mRNA. Hence, while the transgene is necessary for triggering the presence of Cry1Ab toxin, the amount of Cry1Ab toxin produced in the GM plant appears to be influenced by other factors. On the other hand, the systematic increase observed in Cry1Ab concentration following insect-induced stress, regardless of genetic background and outcrossing generation, does suggest that plant metabolic changes of as yet unknown nature can be associated with such a stress.

### Mortality rates of *H. armigera* and *S. littoralis*

#### Brazilian crosses

Mean mortality rates of *H. armigera* larvae fed with maize leaves of different Brazilian crosses containing highly variable concentrations of Cry1Ab toxin (as documented above) ranged between 89.06 and 100% on GM crosses and 17.19–31.25% on the non-GM plants (Additional file 10: Table S9). This led to significant differences in mortality rates between different genetic backgrounds (*Deviance* = 302.77; df = 8; *P* < 0.01), different growing conditions (*Deviance* = 255.28; df = 1; *P* < 0.01) and interactions between different genetic backgrounds and different growing conditions (*Deviance* = 164.93; df = 8; *P* < 0.01).

A detailed analysis of the results showed that, regardless of the different growing conditions, the significant differences in mortality rates of *H. armigera* larvae were between non-GM varieties (ISO and OPV) compared to GM parental maize (*Z* = − 5.136; *P* < 0.01; *Z* = − 5.871; *P* < 0.01). As expected, mortality rates of *H. armigera* larvae were significantly lower on non-GM varieties (ISO and OPV) than on GM parental maize plants. However, there were no significant differences in mortality rates between different genetic backgrounds that produced different Cry1Ab toxin concentrations when compared to GM parental maize (Additional file 11: Table S10).

Regardless of different growing conditions, LSMEANS analysis showed that there was a significant difference in mortality rates of *H. armigera* larvae between non-GM ISO and non-GM OPV maize plants (*Z* = 3.191; df = inf.; *P* < 0.01). Mortality rates were significantly higher when feeding on non-GM ISO plants (25% ± 5.59) than on non-GM OPV plants (9.38% ± 2.42).

**Fig. 10:**
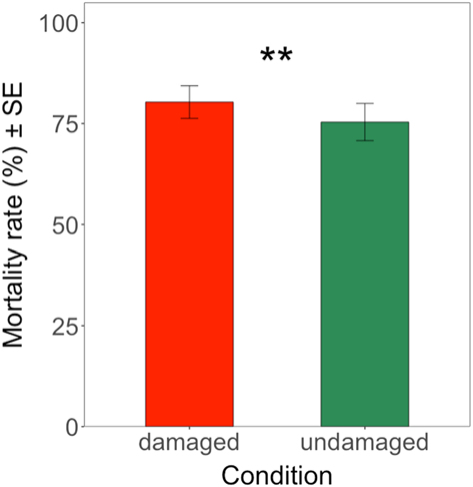
Mean mortality rate (%) of *H. armigera* larvae on damaged and undamaged leaves across all different genetic backgrounds (Brazilian crosses) (including non-GM ISO and non-GM OPV plants). The feeding damage was induced by *H. armigera* larvae. Vertical bars show mean values, and the standard errors (± SE) are indicated as lines. **P < 0.01

Overall, regardless of different genetic backgrounds, mortality rates of *H. armigera* larvae were significantly higher (*Deviance* = 255.28; df = 1; *P* < 0.01) on damaged maize plants (80.33%) than on undamaged maize plants (75.37%) (Fig. 10). A possible explanation may be a combinatorial effect of the increase of the Cry1Ab concentrations and a possible herbivore-induced stimulus which caused damaged maize plants to produce more defence compounds.

**Fig. 11:**
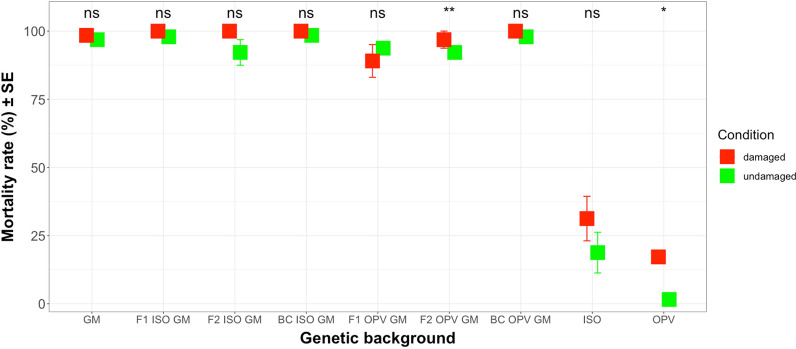
Mean mortality rate (%) of *H. armigera* larvae on damaged and undamaged leaves from different genetic backgrounds (Brazilian crosses). The feeding damage was induced by *H. armigera* larvae. Vertical bars show mean values, and the standard errors (± SE) are indicated as lines. Mortality rate was compared in each different genetic background between damaged and undamaged conditions. ** *P* < 0.01; *P < 0.05; ns: not significant. The p-values reported were not adjusted (LSMEANS analysis)

LSMEANS analysis confirmed that there were significant differences in mortality rates of *H. armigera* when feeding on maize leaves from damaged versus those from undamaged plants of F2 OPV GM (*Z* = 5.316; df = inf.; *P* < 0.01) and non-GM OPV crosses (*Z* = 2.510; df = inf.; *P* < 0.05). Therefore, on F2 OPV GM and on non-GM OPV crosses, the damage inflicted by *H. armigera* larvae caused a slight but statistically significant increase in mortality rates (Fig. 11).

Additionally, statistical analyses of *H. armigera* mortality rates were also carried out excluding the non-GM ISO and non-GM OPV maize plants from the analyses, thus, only including the different genetic backgrounds that produce Cry1Ab protein. The results revealed that there were significant differences in mortality rates between different genetic backgrounds (*Deviance* = 246.97; df = 6; *P* < 0.01), different growing conditions (*Deviance* = 206.58; df = 1; *P* < 0.01) and interactions between different genetic backgrounds and different growing conditions (*Deviance* = 105.96; df = 6; *P* < 0.01). LSMEANS analysis showed that, regardless of the presence of herbivory, these significant differences were due to differences in mortality rates when feeding on GM parental maize versus F1 OPV GM (*Z* = 2.035; df = inf.; *P* < 0.05) and GM parental maize versus F2 OPV GM (*Z* = 4.708; df = inf.; *P* < 0.01). Mortality rates on F1 OPV GM (91.41% ± 3.17) and F2 OPV GM (94.53% ± 1.97) crosses were significantly lower than on GM parental maize (97.66% ± 1.26) although the difference appears to be minor (Additional file 12: Table S11).

**Fig. 12:**
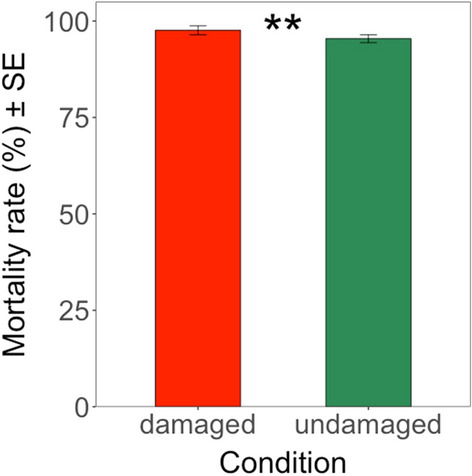
Mean mortality rate (%) of *H. armigera* on damaged and undamaged leaves across all different genetic backgrounds (Brazilian crosses) (excluding non-GM ISO and non-GM OPV plants). The feeding damage was induced by *H. armigera* larvae. Vertical bars show mean values, and the standard errors (± SE) are indicated as lines. **P < 0.01

As described before, regardless of different genetic backgrounds, the mortality rates of *H. armigera* larvae were slightly but significantly (*Deviance* = 206.58; df = 1; *P* < 0.01) higher on damaged maize plants (97.60%) than on undamaged maize plants (95.43%) (Fig. 12).

When the data were pooled and grouped according to ISO and OPV crosses, mean mortality rates of *H. armigera* larvae fed with leaves of the different crosses under damaged conditions ranged between 94.88% in GM OPV crosses and 100% in GM ISO crosses (Additional file 13: Table S12). The results confirmed that there were significant differences in mortality rates between different groups (*Deviance* = 323.38; df = 2; *P* < 0.01), different herbivore induced damage conditions (*Deviance* = 287.51; df = 1; *P* < 0.01) and their interaction effect (*Deviance* = 208.69; df = 2; *P* < 0.05).

LSMEANS analysis revealed that, regardless of different herbivore induced damage conditions, the only significant difference was in mortality rates between GM parental maize and OPV crosses (*Z* = 3.262; df = inf.; *P* < 0.01). Additionally, there were no significant differences between GM parental maize and ISO crosses (*Z* = − 0.017; df = inf.; *P* = 0.987) and between ISO and OPV crosses (*Z* = 0.022; df = inf.; *P* = 0.982). Thus, the mortality rate in OPV crosses (94.60%) was significantly lower than on GM parental maize (97.66%). However, the mortality rate on ISO crosses (98.01%) was similar to those on GM parental maize and, also, similar between ISO and OPV crosses.

Results of the LSMEANS analysis showed that only on OPV crosses, there were significant differences in mortality rates between maize leaves from damaged and undamaged plants (*Z* = 4.098; df = inf.; *P* < 0.01) with the mortality rates being significantly higher on OPV crosses under damaged (94.88%) than under undamaged conditions (94.32%). These results may indicate that OPV maize plants may produce more defense compounds when damaged by herbivores than GM parental plants and ISO crosses. However, additional analyses would need to be done to confirm that.

#### South African crosses

**Fig. 13:**
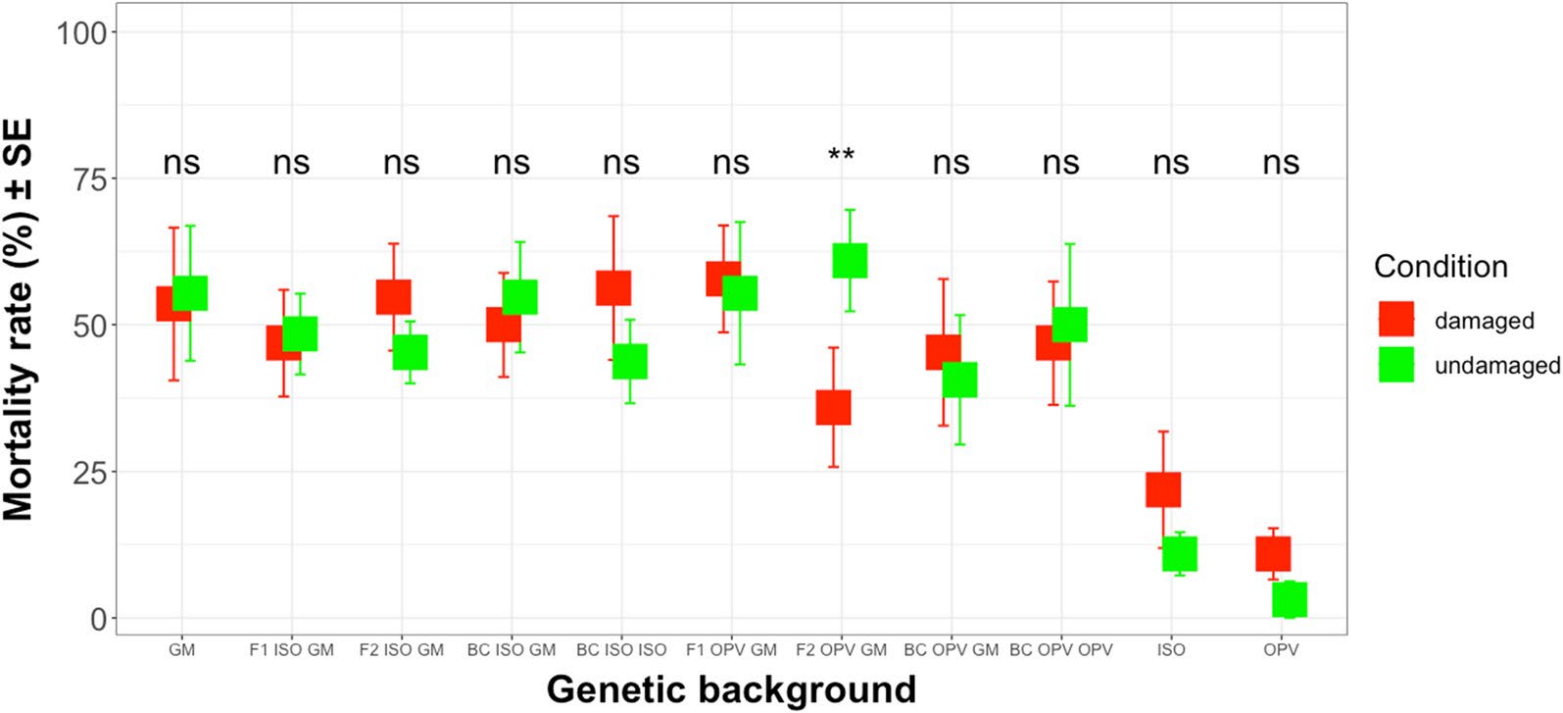
Mean mortality rate (%) of *S. littoralis* larvae on damaged and undamaged leaves from different genetic backgrounds (South Africa crosses). The feeding damage was induced by *S. littoralis* larvae. Vertical bars show mean values, and the standard errors (± SE) are indicated as lines. Mortality rate was compared in each different genetic background between damaged and undamaged conditions. ** *P* < 0.01; ns: not significant. The p-values reported were not adjusted. (LSMEANS analysis)

Mean mortality rates of *S. littoralis* larvae fed with maize leaves of different crosses from South Africa containing different concentrations of Cry1Ab toxin ranged between 35.94 and 57.81% and between 10.94 and 21.88% on non-GM OPV and non-GM ISO plants, respectively (Additional file 10: Table S9). An ANOVA test showed significant differences in mortality rates between different genetic backgrounds (*Deviance* = 462.10; df = 10; *P* < 0.01) as well as the interaction between different genetic backgrounds and different growing conditions (*Deviance* = 319.23; df = 10; *P* < 0.05). However, the test also revealed that, regardless of the different genetic backgrounds, *S. littoralis* damage did not change mortality rates of larvae in the bioassays (*Deviance* = 461.91; df = 1; *P* = 0.670). This result agrees with observations reported above, that Cry1Ab protein concentrations where not influenced by herbivory (Fig. 13).

Results with regards to *S. littoralis* were similar to those found with *H. armigera* on Brazilian crosses. Regardless of different herbivore induced damage conditions, significant differences in mortality rates of *S. littoralis* larvae were observed between non-GM varieties (ISO and OPV) compared to GM parental maize (*Z* = 6.398; df = inf.; *P* < 0.01; *Z* = 6.923; df = inf.; *P* < 0.01). Mortality rates of *S. littoralis* larvae were much lower on non-GM varieties (ISO and OPV) than on GM parental maize plants. However, between different genetic backgrounds producing Cry1Ab toxins, the only significant difference in mortality rates between BC OPV GM backcross and GM parental maize (*Z* = 2.185; df = inf.; *P* < 0.05) with mortality rates of *S. littoralis* larvae being lower on the BC OPV GM backcross (45.31%) than on GM parental maize (53.57%).

Regardless of the different growing conditions, the LSMEANS analysis showed that there was a significant difference in mortality rates of *S. littoralis* larvae when feeding on non-GM ISO versus non-GM OPV maize plants (*Z* = 2.273; df = inf.; *P* < 0.05). Like in the bioassays with *H. armigera* larvae, the mortality rates of *S. littoralis* larvae were also significantly higher on non-GM ISO plants than on non-GM OPV plants.

LSMEANS analyses showed that there were significant differences in mortality rates of *S. littoralis* only when feeding on maize leaves from damaged versus undamaged conditions on F2 OPV GM crosses (*Z* = -2.946; df = inf.; *P* < 0.01). Thus, mortality rates of *S. littoralis* larvae on F2 OPV GM crosses were higher in undamaged conditions (60.94%) than in damaged conditions (35.94%) (Fig. 13).

Statistical analyses of mortality rates of *S. littoralis* larvae were also carried out using data on GM crosses only, excluding non-GM ISO and non-GM OPV maize plants. The ANOVA tests revealed that there were no significant differences in mortality rates between different genetic backgrounds (*Deviance* = 400.55; df = 8; *P* = 0.604), different growing conditions (*Deviance* = 400.48; df = 1; *P* = 0.787) and their interactions (*Deviance* = 277.47; df = 8; *P* = 0.109). Thus, the mortality rates of *S. littoralis* larvae were not influenced by different genetic backgrounds from South Africa, or by herbivore damage to maize plants or by their interactions.

When data were pooled and grouped according to ISO and OPV crosses, mean mortality rates of *S. littoralis* larvae fed with different crosses under damaged condition ranged between 46.48% on OPV crosses and 53.57% on ISO GM parental plants (Additional file 13: Table S12). Results again revealed, again, no significant differences in mortality rates between different groups (*Deviance* = 405.85; df = 2; *P* = 0.579), different herbivore damage conditions (*Deviance* = 405.79; df = 1; *P* = 0.815) and their interactions (*Deviance* = 294.54; df = 2; *P* = 0.292). Summarizing the data in this section, mortality rates of *S. littoralis* larvae were statistically similar between the groups of crosses and GM parental maize (Additional file 13: Table S12). Also, neither herbivore damage nor the interaction between different groups and different growing conditions influence mortality rates of *S. littoralis* larvae.

When feeding on herbivore damaged maize plants, mortality rates of *H. armigera* larvae on Brazilian GM crosses varied twofold (between 50 and 100%). However, when feeding on undamaged maize plants, mortality rates varied 1.6-fold (between 62.5% and 100%). Hence, a Fligner-Killeen test revealed a significant difference in the variances between mortality rates on damaged and undamaged plants (*P* = 0.04), with mortality rates of *H. armigera* on damaged plants varying significantly more than on undamaged maize plants. This result is consistent with the finding for Brazilian crosses in that herbivore damaged maize plants, which also showed higher variability in Cry1Ab protein concentrations. In contrast, mortality rates of *S. littoralis* larvae varied in both growing conditions, between 0 and 100%, with no significant differences in the variances between mortality rates on damaged and undamaged crosses (Fligner-Killeen test, *P* = 0.366).

In summary of this section, the mortality rates of *H. armigera* and *S. littoralis* larvae fed with leaf material of the crosses from different genetic backgrounds that had been exposed to herbivore damage demonstrated that the produced Cry1Ab protein in the crosses and parental GM maize plants were all bioactive. Therefore, no alterations on the post-translational regulatory system were detected.

Although mortality rates of *H. armigera* larvae on Cry1Ab producing plants, regardless of genetic background and outcrossing generation, were fairly high, feeding damage seemed to further increase this mortality rate. On undamaged GM maize crosses, mortality rates were never as high as 100%, while this was the case for four of the seven Cry1Ab crosses exposed to feeding damage. Similarly, also on the non-GM varieties (ISO and OPV), *H. armigera* mortality was higher on damaged than undamaged plants suggesting an increase in defence compounds production and/or expression of defence-related genes. Hence, together, these results may suggest a possible additive effect of Cry1Ab toxin and a possible increase of defence compounds in response to the feeding damage. The effect of these defence compounds on Cry1Ab protein efficacy may be a direct one, where the compounds limit the *H. armigera* larval survival, or an indirect effect, where the compounds interact with the Cry1Ab toxin [66]. However, this was not observed in the experiments with South African crosses and *S. littoralis* larvae, which suggests that this can differ (unpredictably) on a case-by-case basis due to plant genetic background (variety) and pest species.

Some previous studies indicated that defence compounds in crops can alter Bt protein toxicity positively or negatively. Sachs et al. [67] found that pyramiding the CryIAb insecticidal protein with a high-terpenoid trait in cotton could increase the Bt cotton resistance to *Heliothis virescens* (Lepidoptera: Noctuidae) damage. In another study, Mohan et al. [68] demonstrated that a natural defence cysteine protease (Mir1-CP) synergized the effects of the Cry2A toxin against four economically significant lepidopteran pests. Additionally, another study found that tannin improved Cry1Ac toxicity to *H. armigera* [69]. On the other hand, another study revealed a negative effect of plant tannins on Bt protein toxicity [70]. Moreover, Li et al. [71] revealed that quercetin (one of the main flavonoids in cotton) was antagonistic to Cry1Ac toxicity and that its presence in plants reduced its bioactivity agains *H. armigera* pest.

Such evidence in other crops might explain the discrepancies between the results observed here with the two Lepidoptera species, but the complexity of the plant–herbivore response leaves many questions for future research. Upon herbivore attack, constitutive plant defences are complemented by induction of direct defence mechanisms such as production of toxic secondary metabolites [72]. Plant responses to herbivore attack are specific [72, 73], and can be either amplified or suppressed by mechanical damage caused by larval feeding or elicitors present in the oral regurgitant or saliva of herbivores [74–76]. Furthermore, insect salivary secretions can act directly on the plant’s hormone biosynthesis, degradation, transport or signaling pathways to alter phytohormonal balances in the plant [77]. It has also been reported that the production of Bt toxins by maize plants might utilize plant resources that could otherwise be invested in secondary defence compounds [78]. Studies on the effects of herbivore damage to maize seedlings showed rapid responses in terms of secondary compound production after application of larval regurgitant on mechanically damaged plant parts [79]. Ton et al. [80] also showed that undamaged maize plants exposed to volatiles from neighbouring plants infested with larvae of *S. littoralis* showed high levels of priming after infestation by herbivores.

### Correlation between mortality rate and Cry1Ab concentration

**Fig. 14:**
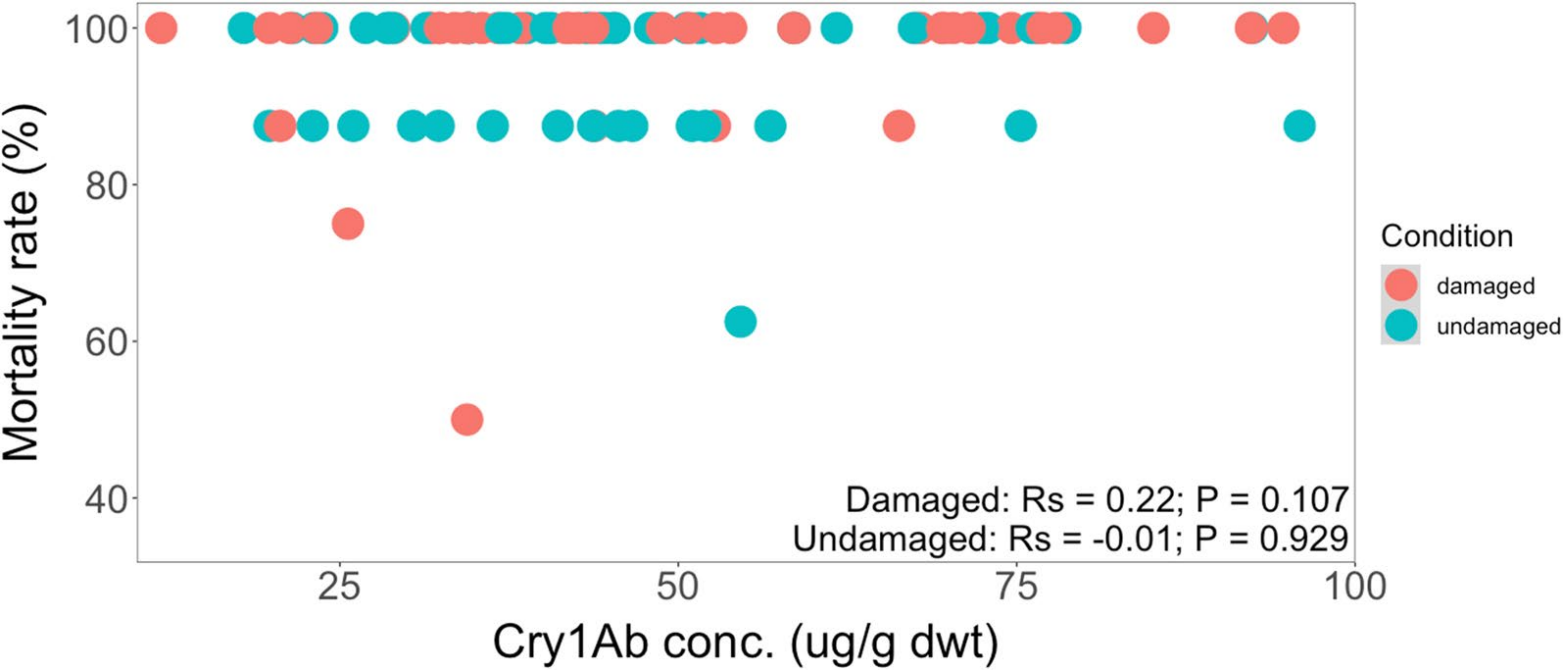
Spearman’s rank correlation coefficient (*Rs*) between mortality rate (%) of the *H. armigera* larvae and Cry1Ab concentration (µg/g dwt) in damaged and undamaged plants across different genetic backgrounds from Brazil. The feeding damage was induced by *H. armigera* larvae. Damaged maize plants (*Rs* = 0.22, *P* = 0.107); undamaged maize plants (*Rs* = − 0.01, *P* = 0.929)

Also here, no statistically significant correlation between mortality rates of *H. armigera* and Cry1Ab concentrations across the different genetic backgrounds from Brazil under herbivore-induced conditions was observed (*Rs* = 0.22, *P* = 0.107) (Fig. 14). There was no correlation between mortality rates of the *H. armigera* larvae and Cry1Ab protein concentrations for undamaged maize plants.

Overall, Cry1Ab protein concentrations produced in all maize plants were high enough to kill more than 85% of the *H. armigera* larvae but rarely 100%.

**Fig. 15:**
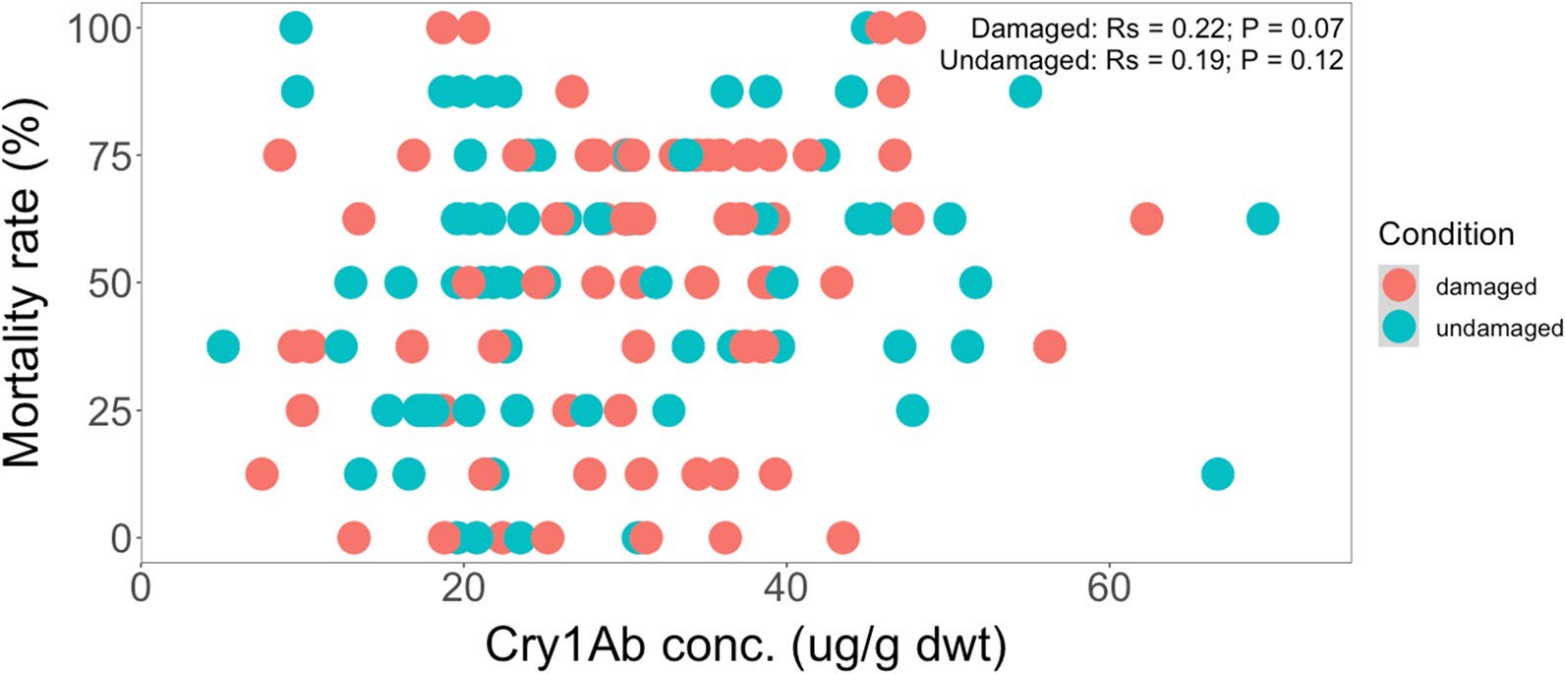
Spearman’s rank correlation coefficient (*Rs*) between the mortality rate (%) of *S. littoralis* larvae and Cry1Ab concentration (µg/g dwt) in damaged and undamaged plants across different genetic backgrounds from South Africa. The feeding damage was induced by *S. littoralis* larvae. Damaged maize plants (*Rs* = 0.22, *P* = 0.07); undamaged maize plants (*Rs* = 0.19, *P* = 0.12)

Likewise, with *S. littoralis*, no statistically significant correlation between mortality rates and Cry1Ab concentrations across different genetic backgrounds from South Africa under herbivore-induced conditions were observed (*Rs* = 0.22, *P* = 0.07) (Fig. 15). Furthermore, there was no correlation between the mortality rates of the *S. littoralis* larvae and the Cry1Ab protein concentrations in undamaged maize plants of South African crosses.

In summary of this section, across all different genetic backgrounds from South Africa and between different growing conditions, the bioassays revealed higher variability in mortality rates of *S. littoralis* larvae (between 0 and 100%) than of *H. armigera* larvae (between 50 and 100%). The higher survival of *S. littoralis* larvae was likely due to its inherently lower susceptibility to Cry1 toxins [81, 82].

Furthermore, a possible explanation for no correlations between the mortality rates and Cry1Ab concentrations are due the high variability in mortality rates of *S. littoralis* and *H. armigera* associated also high variability in Cry1Ab concentrations found in the respective crosses.

Abel and Adamczyk [59] found that V7 maize leaves had higher Cry1Ab concentrations compared to V9 leaves. The leaf tissues of plants of these different plant development stages were fed to larvae of *S. littoralis* and *Diatraea grandiosella* (Lepidoptera: Crambidae), but the authors did not observe a difference in development or an increase in mortality with the tissue that had higher Cry1Ab concentrations. The researchers also stated that they could not explain these results.

On the other hand, in other GM crop studies, researchers found a correlation between the efficacy and the amount of Bt insecticidal protein produced. Adamczyk et al. [83] reported that differences in *Helicoverpa zea* (Lepidoptera: Noctuidae) larval survival and *S. littoralis* larval development correlated with differences in Cry1Ac protein concentration in GM cotton varieties. Mahon et al. [66] and Kranthi el al. [84] also reported a correlation between Cry1Ac protein content and survival rate of *H. armigera* in GM cotton plants. In another study, the researchers revealed that transgenic pea seeds under high temperature produced less alpha-AI-1 (a toxin protein) and this reduction affected the development and survival of *Bruchus pisorum* (Coleoptera: Bruchidae) [85]. Ren et al. [86] also found significant correlation between the mortality rates in *Clostera anachoreta* (Lepidoptera: Notodontidae) and *Lymantria dispar* (Lepidoptera: Lymantriidae) and the Cry1Ac protein content in poplar plants.

## Conclusions

This study has revealed a number of important conclusions: (i) *Cry1Ab* transgene expression levels in GM maize leaves did not change in a systematic fashion when subjected to herbivore-induced damage by two lepidopteran species regardless of the different genetic backgrounds tested, whether from Brazil or South Africa. (ii) Cry1Ab protein concentration was higher in maize plants under herbivore feeding damage by *H. armigera* than in plants under damage-free growing conditions for the crosses obtained from Brazilian GM maize, but not from South Africa. (iii) Regardless of herbivore damage or genetic background of seeds (Brazil or South Africa), *Cry1Ab* transgene transcription levels, as evidenced by mRNA levels, had no clear relation to the specific amount of Cry1Ab protein produced. (iv) Cry1Ab toxin retained its activity in all crosses, and it is expressed in similarly bioactive form in all genetic backgrounds and origins, under undamaged and damaged conditions. (v) South African GM parental maize plants produced higher Cry1Ab concentrations than the respective crosses with ISO and OPV varieties, while the Brazilian GM crosses produced similar concentrations of Cry1Ab toxins compared with their Brazilian parental GM maize variety. (vi) In contrast to the South African crosses, the Brazilian crosses had both significantly higher variability and overall higher concentration of the Cry1Ab protein under herbivore feeding damage, which resulted also in higher variability in mortality rates of the *H. armigera* larvae. (vii) Regardless of presence or absence of herbivore-stress, there was no correlation between the concentration and the efficacy (% mortality) of the Cry1Ab protein in both groups of crosses (Brazil and South Africa).

The qRT-PCR, ELISA and bioassays results suggest that alterations in the path from transgene to mRNA to protein might take place only at the translational level, with a higher production of Cry1Ab protein in the different genetic backgrounds from Brazil under herbivore damage by *H. armigera*. Surprisingly, mRNA transcript level did not seem to influence the levels of Cry1Ab protein production, and it is therefore likely that other metabolic processes, some possibly inducible by insect damage, might have been involved. However, more analyses need to be performed to investigate these processes in more detail at the translational level. The observation that herbivore damage by *S. littoralis* larvae did not affect the Cry1Ab toxin production in different genetic backgrounds from South Africa, shows that it is unpredictable whether or not herbivore damage will trigger additional metabolic changes that affect the efficacy of Bt crops.

To our knowledge, this is the first study investigating the three-way-relationship between Cry1Ab transgene transcription, translation and bioactivity in GM maize plants also considering the context of gene flow to non-GM commercial hybrids and open-pollinating varieties under herbivore damage. We have observed complex and non-trivial effects of genetic context, as well as herbivore stress on the plant, making it difficult to assume a simple relationship between transgene presence and efficacy of the desired insecticidal trait, as is often taken for granted under practical, field conditions. A note of caution is thus warranted not to assume that genetic or ecological diversity will not significantly affect the performance of plants carrying a transgene.

Of particular relevance, risk assessments of resistance evolution in the pests targeted by Bt-transgenic crops should prove more complicated than generally assumed in countries with dual agricultural systems where shared target pests will experience exposure to variable Bt toxin levels, as Bt and non-Bt crosses in various intermixed generations will occur in smallholder fields with highly patchy mixes of genetic backgrounds. Very little is currently known regarding the spread of Bt transgenes in smallholder farmer fields, but given the complexity observed in our results, it can be stated that, at this point, only systematic sampling and monitoring of smallholder farmer’s fields can determine to what degree crossing of non-Bt and Bt maize plants may be affecting their interaction with herbivorous insects. Since the adventitious presence of Bt transgenes in maize fields of smallholder farmers could contribute to a possible acceleration of resistance evolution by herbivorous insects to Bt proteins, not only an assessment of the presence of transgenes in such farming systems will be required in future, but also an awareness that such presence cannot fully reflect the entire range of expression of the transgene and its consequences for the ecology and evolution of target- and non-target insects.

### Supplementary Information

**Additional file 1: Figure S1.** Growth cylinders and maize plants covered by cylinders.

**Additional file 2: Table S1.** Initial number of undamaged and damaged maize seedlings in different genetic backgrounds from Brazil and South Africa.

**Additional file 3: Table S2.** *Cry1Ab* relative transgene transcription levels (mean ±SE) in maize leaves, under damaged and undamaged conditions in different genetic backgrounds from Brazil and South Africa.

**Additional file 4: Table S3.** *Cry1Ab* relative transgene transcription levels (mean ±SE) in maize leaves under damaged and undamaged conditions in different groups from Brazil and South Africa.

**Additional file 5: Table S4.** Cry1Ab concentration (µg/g dwt, mean ±SE) in leaves of maize plants in damaged and undamaged conditions in different genetic backgrounds from Brazil and South Africa.

**Additional file 6: Table S5.** Cry1Ab concentration (µg/g dwt, mean ±SE) in leaves of maize plants in damaged and undamaged conditions across different GM plant groups from South Africa.

**Additional file7: Table S6.** Cry1Ab concentration (µg/g dwt; mean ±SE) in leaves of maize plants, under damaged and undamaged conditions in different groups from Brazil and South Africa.

**Additional file 8: Table S7.** Spearman’s rank correlation between relative transgene transcription levels and Cry1Ab concentration across different genetic backgrounds from Brazil and South Africa under damaged condition.

**Additional file 9 Table S8.** Spearman’s rank correlation between relative transgene transcription levels and Cry1Ab concentration across different groups from Brazil and South Africa, under damaged conditions.

**Additional file 10: Table S9.** Mortality rates (% ±SE) of *H. armigera *and *S. littoralis* larvae fed on maize leaves from different genetic backgrounds under damaged and undamaged growing conditions (n = number of leaves).

**Additional file 11: Table S10.** Multiple comparisons of means (Dunnett’s method) in the control group (GM parental maize) from Brazil, including non-GM ISO and non-GM OPV plants. The p-values reported were adjusted by single-step method.

**Additional file 12: Table S11.** Multiple comparisons of means (Dunnett’s method) in the control group (GM parental maize) from Brazil, excluding non-GM ISO and non-GM OPV plants. The p-values reported were adjusted by the single-step method.

**Additional file 13: Table S12.** Mortality rates (%, mean and standard error ±SE) of in different groups and in damaged and undamaged growing conditions. Number of maize leaves and of *H. armigera* and *S. littoralis* larvae utilized.

## Data Availability

The datasets used and/or analyzed during the current study are available from the corresponding author on reasonable request.

## Abbreviations

Bt: *Bacillus thuringiensis*
cDNA: Complementary DNA
DNA: Deoxyribonucleic acid
DWT: Dry weight tissue
ELISA: Enzyme-linked immunosorbent assay
GM: Genetically modified
L:D: Light:Dark
mRNA: Messenger RNA
N°: Number
qRT-PCR: Real-time polymerase chain reaction
RNA: Ribonucleic acid
*Rs*: Spearman rank correlation coefficient
SE: Standard error
