# Supplementary material for: Effect of herbivore stress on transgene behaviour in maize crosses with different genetic backgrounds: cry1Ab transgene transcription, insecticidal protein expression and bioactivity against insect pests
Source: Environ Sci Eur. 2023 Nov 28;35(1):106. doi: 10.1186/s12302-023-00815-3 (PMC10684648; doi:10.1186/s12302-023-00815-3)
Supplement: Supplementary file 1 — Additional file 1: Figure S1. Growth cylinders and maize plants covered by cylinders. [file 12302_2023_815_MOESM1_ESM.pdf]

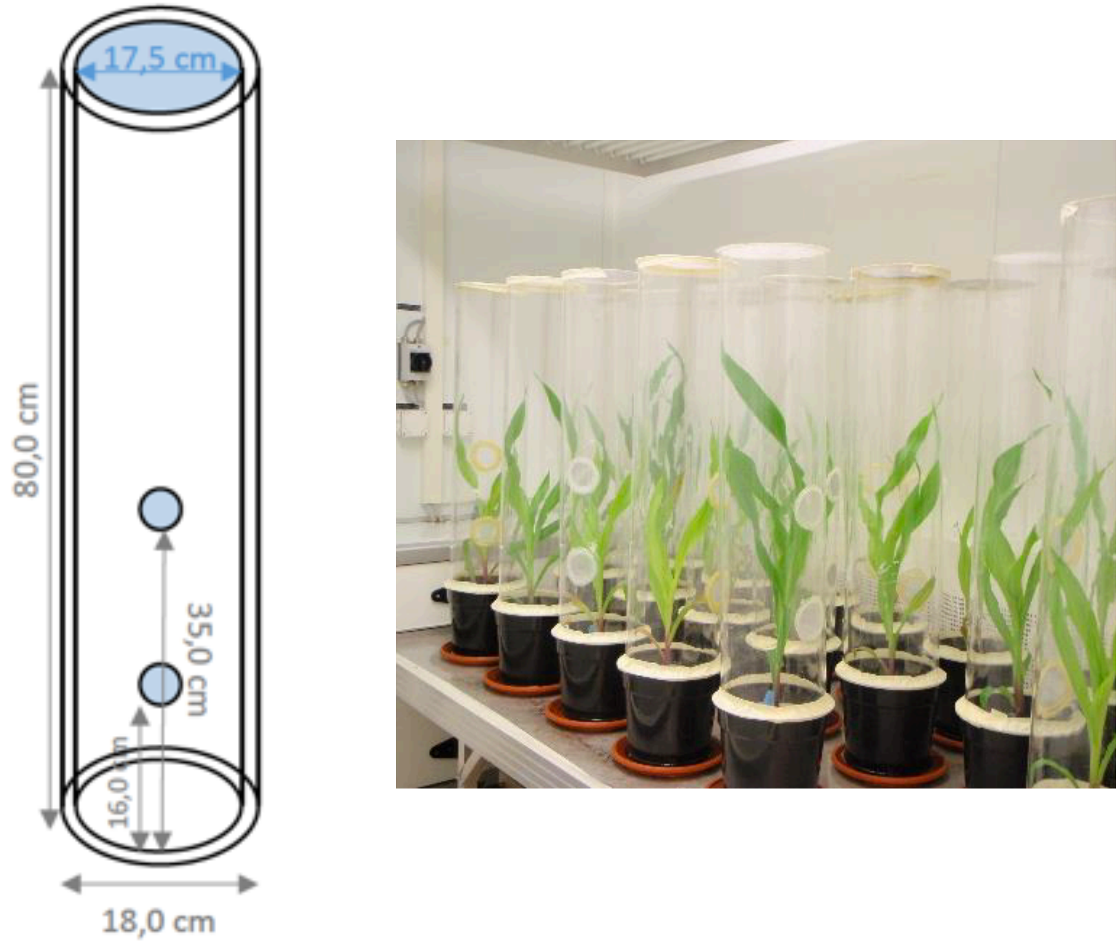

On left: design of the used PLEXIGLAS® cylinder; on right: maize plants covered by cylinders (Figure credit: Wiget M, (2015) Effect of *Spodoptera littoralis* herbivory on defense mechanisms of Bt maize. PEG, ETH/Zurich).
