# Supplementary material for: Effect of herbivore stress on transgene behaviour in maize crosses with different genetic backgrounds: cry1Ab transgene transcription, insecticidal protein expression and bioactivity against insect pests
Source: Environ Sci Eur. 2023 Nov 28;35(1):106. doi: 10.1186/s12302-023-00815-3 (PMC10684648; doi:10.1186/s12302-023-00815-3)
Supplement: Supplementary file 2 — Additional file 2: Table S1. Initial number of undamaged and damaged maize seedlings in different genetic backgrounds from Brazil and South Africa. [file 12302_2023_815_MOESM2_ESM.pdf]

Genetic background	N° of seedlings			
	undamaged		damaged	
	Brazil	South Africa	Brazil	South Africa
GM	8	7	8	7
F1 ISO GM	6	7	6	8
F2 ISO GM	8	8	8	8
BC ISO GM	8	8	8	8
BC ISO ISO	-	8	-	8
F1 OPV GM	8	8	8	8
F2 OPV GM	8	8	8	8
BC OPV GM	6	8	6	8
BC OPV OPV	-	8	-	8
ISO	8	8	8	8
OPV	8	8	8	8
**Total**	**68**	**86**	**68**	**87**
