# Supplementary material for: Effect of herbivore stress on transgene behaviour in maize crosses with different genetic backgrounds: cry1Ab transgene transcription, insecticidal protein expression and bioactivity against insect pests
Source: Environ Sci Eur. 2023 Nov 28;35(1):106. doi: 10.1186/s12302-023-00815-3 (PMC10684648; doi:10.1186/s12302-023-00815-3)
Supplement: Supplementary file 3 — Additional file 3: Table S2. Cry1Ab relative transgene transcription levels (mean ±SE) in maize leaves, under damaged and undamaged conditions in different genetic backgrounds from Brazil and South Africa. [file 12302_2023_815_MOESM3_ESM.pdf]

Genetic background	Brazil				South Africa			
	undamaged		damaged		undamaged		damaged	
	N° of plants	Rel. transgene expression ± SE	N° of plants	Rel. transgene expression ± SE	N° of plants	Rel. transgene expression ± SE	N° of plants	Rel. transgene expression ± SE
GM	8	1.57 ± 0.23	8	1.17 ± 0.11	6	1.25 ± 0.13	6	1.17 ± 0.16
F1 ISO GM	5	1.64 ± 0.16	6	0.59 ± 0.19	7	0.90 ± 0.19	8	1.19 ± 0.17
F2 ISO GM	8	1.35 ± 0.19	8	1.01 ± 0.19	8	1.29 ± 0.21	6	1.34 ± 0.17
BC ISO GM	7	0.75 ± 0.03	8	1.43 ± 0.19	8	1.21 ± 0.19	8	1.22 ± 0.19
BC ISO ISO	-	-	-	-	8	0.78 ± 0.17	8	0.98 ± 0.14
F1 OPV GM	8	1.02 ± 0.18	8	0.89 ± 0.17	8	1.03 ± 0.14	8	1.24 ± 0.13
F2 OPV GM	8	1.12 ± 0.21	8	0.86 ± 0.28	6	1.45 ± 0.28	8	1.51 ± 0.23
BC OPV GM	6	1.30 ± 0.30	6	1.36 ± 0.27	8	0.79 ± 0.18	6	0.80 ± 0.15
BC OPV OPV	-	-	-	-	8	0.70 ± 0.09	8	1.09 ± 0.23
