# Supplementary material for: Effect of herbivore stress on transgene behaviour in maize crosses with different genetic backgrounds: cry1Ab transgene transcription, insecticidal protein expression and bioactivity against insect pests
Source: Environ Sci Eur. 2023 Nov 28;35(1):106. doi: 10.1186/s12302-023-00815-3 (PMC10684648; doi:10.1186/s12302-023-00815-3)
Supplement: Supplementary file 4 — Additional file 4: Table S3. Cry1Ab relative transgene transcription levels (mean ±SE) in maize leaves under damaged and undamaged conditions in different groups from Brazil and South Africa. [file 12302_2023_815_MOESM4_ESM.pdf]

Group	Brazil				South Africa			
	undamaged		damaged		undamaged		damaged	
	N° of plants	Rel. transgene expression ± SE	N° of plants	Rel. transgene expression ± SE	N° of plants	Rel. transgene expression ± SE	N° of plants	Rel. transgene expression ± SE
GM	8	1.57 ± 0.23	8	1.17 ± 0.11	6	1.25 ± 0.13	6	1.17 ± 0.16
ISO crosses	20	1.21 ± 0.11	22	1.05 ± 0.13	31	1.05 ± 0.10	30	1.17 ± 0.08
OPV crosses	22	1.13 ± 0.13	25	1.00 ± 0.14	30	0.96 ± 0.10	30	1.19 ± 0.10
