# Supplementary material for: Effect of herbivore stress on transgene behaviour in maize crosses with different genetic backgrounds: cry1Ab transgene transcription, insecticidal protein expression and bioactivity against insect pests
Source: Environ Sci Eur. 2023 Nov 28;35(1):106. doi: 10.1186/s12302-023-00815-3 (PMC10684648; doi:10.1186/s12302-023-00815-3)
Supplement: Supplementary file 5 — Additional file 5: Table S4. Cry1Ab concentration (µg/g dwt, mean ±SE) in leaves of maize plants in damaged and undamaged conditions in different genetic backgrounds from Brazil and South Africa. [file 12302_2023_815_MOESM5_ESM.pdf]

Genetic background	Brazil				South Africa			
	undamaged		damaged		undamaged		damaged	
	N° of plants	Cry1Ab concentration (ug/g dwt) ± SE	N° of plants	Cry1Ab concentration (ug/g dwt) ± SE	N° of plants	Cry1Ab concentration (ug/g dwt) ± SE	N° of plants	Cry1Ab concentration (ug/g dwt) ± SE
GM	8	42.78 ± 5.68	8	54.29 ± 7.69	6	40.85 ± 4.57	7	37.30 ± 3.87
F1 ISO GM	5	48.26 ± 9.00	6	50.01 ± 11.30	7	27.47 ± 5.05	8	27.20 ± 2.04
F2 ISO GM	6	35.12 ± 6.21	8	63.39 ± 10.54	8	24.45 ± 4.17	7	25.80 ± 5.28
BC ISO GM	8	45.59 ± 6.74	8	75.08 ± 8.03	7	30.54 ± 4.60	8	33.28 ± 5.22
BC ISO ISO	-	-	-	-	8	28.18 ± 4.10	8	29.89 ± 2.76
F1 OPV GM	7	41.27 ± 3.97	8	41.76 ± 6.60	8	20.40 ± 2.90	8	28.69 ± 3.62
F2 OPV GM	8	48.48± 4.87	8	41.96 ± 9.70	8	28.35 ± 5.39	8	23.29 ± 4.65
BC OPV GM	5	39.78 ± 9.24	6	66.53 ± 11.23	8	25.39 ± 3.53	8	38.25 ± 3.34
BC OPV OPV	-	-	-	-	8	25.35 ± 2.17	8	30.48 ± 3.36
