# Supplementary material for: Effect of herbivore stress on transgene behaviour in maize crosses with different genetic backgrounds: cry1Ab transgene transcription, insecticidal protein expression and bioactivity against insect pests
Source: Environ Sci Eur. 2023 Nov 28;35(1):106. doi: 10.1186/s12302-023-00815-3 (PMC10684648; doi:10.1186/s12302-023-00815-3)
Supplement: Supplementary file 6 — Additional file 6: Table S5. Cry1Ab concentration (µg/g dwt, mean ±SE) in leaves of maize plants in damaged and undamaged conditions across different GM plant groups from South Africa. [file 12302_2023_815_MOESM6_ESM.pdf]

Condition	N° of plants	Cry1Ab concentration (µg/g dwt) ± SE
Damaged	70	30.43 ± 1.35
Undamaged	66	27.69 ± 1.44
