# Supplementary material for: Effect of herbivore stress on transgene behaviour in maize crosses with different genetic backgrounds: cry1Ab transgene transcription, insecticidal protein expression and bioactivity against insect pests
Source: Environ Sci Eur. 2023 Nov 28;35(1):106. doi: 10.1186/s12302-023-00815-3 (PMC10684648; doi:10.1186/s12302-023-00815-3)
Supplement: Supplementary file 7 — Additional file7: Table S6. Cry1Ab concentration (µg/g dwt; mean ±SE) in leaves of maize plants, under damaged and undamaged conditions in different groups from Brazil and South Africa. [file 12302_2023_815_MOESM7_ESM.pdf]

Group	Brazil				South Africa			
	undamaged		damaged		undamaged		damaged	
	**N° of plants**	**Cry1Ab concentration (ug/g dwt) ± SE**	**N° of plants**	**Cry1Ab concentration (ug/g dwt) ± SE**	**N° of plants**	**Cry1Ab concentration (ug/g dwt) ± SE**	**N° of plants**	**Cry1Ab concentration (ug/g dwt) ± SE**
GM	8	42.78 ± 5.68	8	54.29 ± 7.69	6	40.85 ± 4.57	7	37.30 ± 3.87
ISO crosses	19	42.98 ± 4.15	22	64.00 ± 5.85	30	27.57 ± 2.15	31	29.14 ± 1.97
OPV crosses	20	43.78 ± 3.24	22	48.59 ± 5.53	30	25.17 ± 1.88	32	30.18 ± 2.04
