# Supplementary material for: Effect of herbivore stress on transgene behaviour in maize crosses with different genetic backgrounds: cry1Ab transgene transcription, insecticidal protein expression and bioactivity against insect pests
Source: Environ Sci Eur. 2023 Nov 28;35(1):106. doi: 10.1186/s12302-023-00815-3 (PMC10684648; doi:10.1186/s12302-023-00815-3)
Supplement: Supplementary file 8 — Additional file 8: Table S7. Spearman’s rank correlation between relative transgene transcription levels and Cry1Ab concentration across different genetic backgrounds from Brazil and South Africa under damaged condition. [file 12302_2023_815_MOESM8_ESM.pdf]

Genetic background	P		Spearman's correlation (Rs)	
	Brazil	South Africa	Brazil	South Africa
GM	0.10	0.24	-0.64	0.60
F1 ISO GM	0.33	0.66	0.80	-0.19
F2 ISO GM	0.43	0.56	0.33	0.29
BC ISO GM	0.93	0.30	0.05	-0.54
BC ISO ISO	-	0.39	-	0.36
F1 OPV GM	0.43	0.58	0.33	0.24
F2 OPV GM	0.4	0.43	0.39	0.33
BC OPV GM	1.00	0.22	0.03	0.50
BC OPV OPV	-	0.70	-	0.17
