# Supplementary material for: Effect of herbivore stress on transgene behaviour in maize crosses with different genetic backgrounds: cry1Ab transgene transcription, insecticidal protein expression and bioactivity against insect pests
Source: Environ Sci Eur. 2023 Nov 28;35(1):106. doi: 10.1186/s12302-023-00815-3 (PMC10684648; doi:10.1186/s12302-023-00815-3)
Supplement: Supplementary file 9 — Additional file 9 Table S8. Spearman’s rank correlation between relative transgene transcription levels and Cry1Ab concentration across different groups from Brazil and South Africa, under damaged conditions. [file 12302_2023_815_MOESM9_ESM.pdf]

Group	P		Spearman's correlation (Rs)	
	**Brazil**	**South Africa**	**Brazil**	**South Africa**
GM	0.10	0.24	-0.64	0.60
ISO crosses	0.23	0.94	0.27	0.01
OPV crosses	0.21	0.88	0.28	0.03
