# Supplementary material for: Effect of herbivore stress on transgene behaviour in maize crosses with different genetic backgrounds: cry1Ab transgene transcription, insecticidal protein expression and bioactivity against insect pests
Source: Environ Sci Eur. 2023 Nov 28;35(1):106. doi: 10.1186/s12302-023-00815-3 (PMC10684648; doi:10.1186/s12302-023-00815-3)
Supplement: Supplementary file 10 — Additional file 10: Table S9. Mortality rates (% ±SE) of H. armigera and S. littoralis larvae fed on maize leaves from different genetic backgrounds under damaged and undamaged growing conditions (n = number of leaves). [file 12302_2023_815_MOESM10_ESM.pdf]

Genetic background	Brazil						South Africa					
	undamaged			damaged			undamaged			damaged		
	N° of leaves	N° of larvae	Mort. rate (%) ± SE	N° of leaves	N° of larvae	Mort. rate (%) ± SE	N° of leaves	N° of larvae	Mort. rate (%) ± SE	N° of leaves	N° of larvae	Mort. rate (%) ± SE
GM	8	64	96.88± 2.05	8	64	98.44 ± 1.56	7	56	55.36 ± 11.53	7	56	53.57 ± 13.00
F1 ISO GM	6	48	97.92 ± 2.08	6	48	100 ± 0	8	64	48.44 ± 6.86	8	64	46.88 ± 9.07
F2 ISO GM	8	64	92.19 ± 4.69	8	64	100 ± 0	8	64	45.31 ± 5.25	8	64	54.69 ± 9.13
BC ISO GM	8	64	98.44 ± 1.56	8	64	100 ± 0	8	64	54.69 ± 9.43	8	64	50.00 ± 8.84
BC ISO ISO	-	-	-	-	-	-	8	64	43.75 ± 7.09	8	64	56.25 ± 12.28
F1 OPV GM	8	64	93.75 ± 2.36	8	64	89.06 ± 5.99	7	56	55.36 ± 12.16	8	64	57.81 ± 9.13
F2 OPV GM	8	64	92.19 ± 2.29	8	64	96.88 ± 3.13	8	64	60.94 ± 8.66	8	64	35.94 ± 10.14
BC OPV GM	6	48	97.92 ± 2.08	6	48	100 ± 0	8	64	40.63 ± 11.02	8	64	45.31 ± 12.49
BC OPV OPV	-	-	-	-	-	-	8	64	50.00 ± 13.77	8	64	46.88 ± 10.50
ISO	8	64	18.75 ± 7.47	8	64	31.25 ± 8.18	8	64	10.94 ± 3.69	8	64	21.88 ± 9.95
OPV	8	64	1.56 ± 1.56	8	64	17.19 ± 2.29	8	64	3.13 ± 3.13	8	64	10.94 ± 4.38
