# Supplementary material for: Effect of herbivore stress on transgene behaviour in maize crosses with different genetic backgrounds: cry1Ab transgene transcription, insecticidal protein expression and bioactivity against insect pests
Source: Environ Sci Eur. 2023 Nov 28;35(1):106. doi: 10.1186/s12302-023-00815-3 (PMC10684648; doi:10.1186/s12302-023-00815-3)
Supplement: Supplementary file 11 — Additional file 11: Table S10. Multiple comparisons of means (Dunnett’s method) in the control group (GM parental maize) from Brazil, including non-GM ISO and non-GM OPV plants. The p-values reported were adjusted by single-step method. [file 12302_2023_815_MOESM11_ESM.pdf]

| Comparison | Estimate | Std. Error | z value | Pr (>z) |
|---|---|---|---|---|
| F1 ISO GM - GM | 16.444 | 2802.469 | 0.006 | 1.000 |
| F2 ISO GM - GM | 16.631 | 2449.339 | 0.007 | 1.000 |
| BC ISO GM - GM | 16.631 | 2449.339 | 0.007 | 1.000 |
| F1 OPV GM - GM | -2.100 | 1.093 | -1.922 | 0.288 |
| F2 OPV GM - GM | -1.919 | 1.104 | -1.739 | 0.401 |
| BC OPV GM- GM | 16.354 | 2813.837 | 0.006 | 1.000 |
| ISO - GM | -5.463 | 1.064 | -5.136 | **<0.001** |
| OPV - GM | -6.370 | 1.085 | -5.871 | **<0.001** |
