# Supplementary material for: Effect of herbivore stress on transgene behaviour in maize crosses with different genetic backgrounds: cry1Ab transgene transcription, insecticidal protein expression and bioactivity against insect pests
Source: Environ Sci Eur. 2023 Nov 28;35(1):106. doi: 10.1186/s12302-023-00815-3 (PMC10684648; doi:10.1186/s12302-023-00815-3)
Supplement: Supplementary file 12 — Additional file 12: Table S11. Multiple comparisons of means (Dunnett’s method) in the control group (GM parental maize) from Brazil, excluding non-GM ISO and non-GM OPV plants. The p-values reported were adjusted by the single-step method. [file 12302_2023_815_MOESM12_ESM.pdf]

**Comparison**	**Estimate**	**Std. Error**	**z ratio**	**p.value**
F1 ISO GM - GM	-8.225	1285.862	-0.006	0.995
F2 ISO GM - GM	-6.902	1146.677	-0.006	0.995
BC ISO GM - GM	-8.614	1146.677	-0.008	0.994
F1 OPV GM - GM	1.445	0.710	2.035	**0.042**
F2 OPV GM - GM	3.270	0.694	4.708	**<0.0001**
BC OPV GM- GM	-8.089	1295.140	-0.006	0.995
