# Supplementary material for: Effect of herbivore stress on transgene behaviour in maize crosses with different genetic backgrounds: cry1Ab transgene transcription, insecticidal protein expression and bioactivity against insect pests
Source: Environ Sci Eur. 2023 Nov 28;35(1):106. doi: 10.1186/s12302-023-00815-3 (PMC10684648; doi:10.1186/s12302-023-00815-3)
Supplement: Supplementary file 13 — Additional file 13: Table S12. Mortality rates (%, mean and standard error ±SE) of in different groups and in damaged and undamaged growing conditions. Number of maize leaves and of H. armigera and S. littoralis larvae utilized. [file 12302_2023_815_MOESM13_ESM.pdf]

Group	Brazil						South Africa					
	undamaged			damaged			undamaged			damaged		
	N° of leaves	N° of larvae	Mort. rate (%) ± SE	N° of leaves	N° of larvae	Mort. rate (%) ± SE	N° of leaves	N° of larvae	Mort. rate (%) ± SE	N° of leaves	N° of larvae	Mort. rate (%) ± SE
GM	8	64	96.88 ± 2.05	8	64	98.44 ± 1.56	7	56	55.36 ± 11.53	7	56	53.57 ± 13.00
ISO crosses	22	176	96.02 ± 1.91	22	176	100 ± 0	32	256	48.05 ± 3.55	32	256	51.95 ± 4.77
OPV crosses	22	176	94.32 ± 1.36	22	176	94.88 ± 2.56	31	248	51.61 ± 5.64	32	256	46.48 ± 5.24
